# Single-cell profiling reveals periosteal signatures of impaired periosteal cells proliferation in a drill-hole model of type 2 diabetes

**DOI:** 10.1186/s12964-025-02349-y

**Published:** 2025-08-12

**Authors:** Xing Ji, Jiahao Luo, Yangxun He, Xinhua Hu, Taotao Xu, Yuanlong Wang, Sijun Pan, Jiali Yao, Weiwei Hu, Ximei Wu

**Affiliations:** 1 Department of Pharmacology, Zhejiang University School of Medicine, 866 Yuhangtang Road, Hangzhou, 310058 China; 2 Department of Pharmacology, Hangzhou City University School of Medicine, 51 Huzhou Street, Hangzhou, 310015 China; 3 Department of Clinical Pharmacology, The Second Affiliated Hospital of Zhejiang University School of Medicine, 88 Jiefang Road, Hangzhou, 310009 China; 4 Department of Orthopedics Surgery, The First Affiliated Hospital of Zhejiang Chinese Medical University, 54 Youdian Road, Hangzhou, 310006 China; 5 Anji People’s Hospital, Affiliated Anji Hospital, School of Medicine, Hangzhou City University, Hangzhou, 310015 China; 6 Shulan International Medical College, Zhejiang Shuren University, 8 Shuren Street, Hangzhou, 310015 China

**Keywords:** Periosteum, T2DM, Delayed fracture healing, Fibrinogen-like Protein 2, ScRNA-seq

## Abstract

Type 2 diabetes mellitus (T2DM) is associated with an elevated fracture risk and impaired healing, but the periosteum’s role in delayed repair remains unclear. In db/db mice, both trabecular and cortical bone mass were reduced, with single-cell RNA sequencing revealing downregulation of the Wnt pathway in osteogenic periosteal cells, which is critical for maintaining cortical bone. Transcriptomic analysis of periosteal cells from humans with T2DM further underscored the evolutionary conservation of osteogenic properties. A comprehensive atlas of periosteal cells under WT and T2DM conditions, pre- and post-fracture, identified induced fibrogenic cells as essential for fracture repair. Further analysis confirmed that induced fibrogenic cells contribute to both intramembranous and endochondral ossification. Importantly, we identified Fibrinogen-like Protein 2 (FGL2), expressed by fibro-adipogenic progenitors (FAPs) and periosteal cells, as a key factor hindering healing by suppressing periosteal proliferation through mitochondrial regulation via the mTORC1 pathway. These findings highlight the periosteal heterogeneity and dynamics involved in delayed fracture healing in T2DM.

**Supplementary Information:**

The online version contains supplementary material available at 10.1186/s12964-025-02349-y.

## Introduction

Type 2 Diabetes Mellitus (T2DM) confers a substantial increase in the susceptibility to bone healing process complications, often culminating in delayed healing or nonunion [1–3]. Despite this substantial health burden, the current standard treatment for patients with slow or incomplete bone healing remains bone grafting, utilizing either an autograft or an allograft [4]. Nevertheless, the efficacy of bone grafting is constrained by the availability of suitable autogenous or allogenous osseous material and the potential for iatrogenic compli cations, including hematoma and aberrant bone remodeling [5, 6].

Traditionally, the cambium layer of the periosteum and bone marrow stromal cells have been regarded as crucial for fracture healing. Though the role of bone marrow stromal cells in fracture healing has been extensively studied, and periosteal cells have been increasingly recognized as key contributors to this process, their heterogeneity and functional alterations under T2DM conditions remain unexplored at single-cell resolution [7, 8]. The periosteum is composed of two layers: the fibrous layer and the cambium layer. Recent advances in Single-cell RNA sequencing (sc-RNA seq) and lineage tracing have underscored the significant contributions of periosteal cells, particularly those from the fibrous layer to the fracture repair process [9]. However, under conditions of T2DM, the specific role of the periosteum, particularly in the delayed healing of fractures, remains poorly understood.

Secreted growth factors, such as fibroblast growth factors (FGFs), platelet-derived growth factors (PDGFs), transforming growth factor-betas (TGF-βs), vascular endothelial growth factor (VEGF), bone morphogenetic proteins (BMPs) and Wnt ligands, are key regulators of Mesenchymal stem cell (MSC) differentiation and are essential in promoting bone formation during bone healing [10–14]. When recombinant human BMP2 (rhBMP2) is added to stabilized fractures, it increases cartilage and overall volume but does not lead to a corresponding increase in intramembranous bone formation [15]. The potential existence of other factors secreted during fracture healing in T2DM that may contribute to the healing process has yet to be fully explored. Osteoblast differentiation depends on glycolysis [16]. BMP and WNT-LRP5 signaling drives bone formation by directly reprogramming glucose metabolism [17, 18]. Recently our studies have shown that activating glycolysis in the osteoblast lineage can reverse osteoporotic phenotypes in both type 1 diabetes mellitus (T1DM) and T2DM [19, 20].

In this study, we performed drill hole surgery to generate a bone repair mouse model by intramembranous ossification instead of by endochondral ossification occurring in tibia fractures [21]. The results indicate that leptin receptor-deficient (db/db) mice exhibit osteoporosis and delayed bone healing. This osteoporotic phenotype was confirmed by micro-CT and histomorphometric analyses, and impaired bone repair was further validated using the drill-hole injury model, with a significant reduction in bone formation and periosteal cell proliferation following fractures.

Sc-RNA seq of the periosteal cells from both db/+ and db/db mice before and 7 days after fracture revealed injury-induced fibrogenic features following the drill hole surgery. Notably, Fibrinogen-like protein 2 (FGL2), a secreted protein initially identified in immune cells such as macrophages and T cells [22, 23], was found to be expressed in the periosteum and at the surgical site. In vitro, high-glucose conditions suppressed the proliferative effect of FGL2 on periosteal cells, suggesting that impaired FGL2-mediated signaling may contribute to delayed fracture healing in the diabetic setting.

## Material and methods

### Mice

All mice work was conducted with the approval of the Institutional Animal Care and Use Committee at Zhejiang University. Male BKS-LeprKO/KO (db/db) mice and heterzygous db/+ mice on a C57BKS background, as well as C57BL/6J mice, were acquired from GemPharmatech Co., Ltd. The db/db mice served as the diabetic experimental group, while the db/+ mice were used as the non-diabetic control group. Housing conditions included pairing two db/db mice and four db/+ mice per cage. All mice were provided with commercial diet and distilled water. The environmental conditions were maintained at a controlled temperature of 23 ± 1 °C, with a 12-h light/12-h dark cycle and relative humidity kept between 50 and 60%.

### Isolation and in vitro culture of periosteal cells

12-week-old male C57BL/6J mice were euthanized using CO_2_ inhalation followed by cervical dislocation, and periosteal cells were isolated using a previously described method [19]. After removing the majority of muscle and connective tissues, the femoral and tibial epiphyses were embedded in 5% low melting point agarose (Yeasen, 10214ES08). The bones were subsequently digested in alpha Minimum Essential Medium (αMEM) supplemented with 3 mg/ml collagenase (Worthington, LS004176) and 4 mg/ml dispase (Sigma-Aldrich, D4693). The digestion was performed in four sequential rounds, each with agitation at 37 °C for 15 min. The first digestion fraction was discarded, and the remaining three fractions were pooled. The pooled cell suspension was passed through a 70 μm cell strainer (Corning) to remove debris, then centrifuged at 300 × g for 5 min at room temperature. The resulting cell pellet was resuspended in growth medium and seeded into culture plates for downstream experiments. Once the periosteal cells reached approximately 90% confluence, experiments were initiated using cells plated at a density of 1.5 × 10^4^ cells/cm^2^. Periosteal cells in the diabetic group were cultured in a high glucose medium (25.5 mM/L) to maintain a sugar-rich environment. Recombinant mouse FGL2 proteins (MedChem Express, HY-P70090) used in the in vitro cell treatments.

### CRISPR-Cas9 plasmid construction and transformation

Single-guide RNAs (sgRNAs) targeting Raptor and a non-targeting control (sgControl) were designed, and the top-ranking sequences were synthesized as oligonucleotide pairs (Sangon Biotech, Shanghai, China) (Supplementary Table S1). Oligos were annealed and phosphorylated using T4 PNK, then diluted 1:200 for ligation. Annealed products were cloned into BsmBI-digested LentiCRISPRv2 vector (Addgene #52961) using a combined digestion-ligation reaction with Esp3I and T7 ligase. The reaction was cycled between 37 °C and 20 °C for 15 cycles. Ligation products were transformed into E. coli Stbl3 competent cells by heat shock. After recovery in LB medium, bacteria were plated on LB agar containing 100 µg/mL ampicillin. Colonies were screened by Sanger sequencing (Youkang Biosciences, Hangzhou, China) to confirm sgRNA insertion.

### Lentivirus preparation and infection

The construction of the CRISPR-Cas9 plasmid has been described previously [24]. sgRNA sequences were designed as detailed. Periosteal cells were plated at a density of 1.5 × 10^4^ cells/cm^2^ and cultured overnight. Lentiviral supernatant preparation and infection procedures were carried out following established methods [25].

### RNA isolation, cDNA preparation, and real-time quantitative PCR

Cell lysates were prepared using RNAiso Plus (Takara, 9109) following the manufacturer’s instructions. Reverse transcription was carried out using HiScript II qRT SuperMix (Vazyme, B2291JAA) according to the manufacturer’s protocol. The CFX96™ Real-Time PCR Detection System (Bio-Rad) and ChamQ Universal qPCR Master Mix (Vazyme, Q311-02) were utilized to measure mRNA expression levels of Raptor and β-actin. Primers used for qPCR are listed (Supplementary Table S1). Relative gene expression differences were quantified using the 2^−ΔΔ*Ct*^ method.

### Protein extraction and western blot assay

Periosteal cells were plated and cultured for 48 h with or without FGL2 recombinant proteins. The primary antibodies used included anti-S6 (CST, #2217), anti-phospho-S6 (CST, 2211), and anti-α-tubulin (Huabio, ER130905). Secondary antibodies were IRDye® 680RD (LI-COR, 926–68070) and IRDye® 800CW (LI-COR, 926–32211). α-Tubulin (CST, #2125S) was used as the cytosolic internal reference. Protein bands were imaged using the Odyssey® DLX system (LI-COR), and protein abundance was quantified and normalized using Image Studio™ Software (version 5.2).

### Cell proliferation assays

Periosteal cells were plated at a density of 3 × 10^3^ cells/cm^2^ in 96-well plates and cultured with or without FGL2 recombinant proteins for durations of 48 h, 72 h and 96 h. The culture medium was refreshed daily. Cell proliferation was assessed using both CCK-8 methods, according to the manufacturer’s instructions (Beyotime, C0088).

### Glucose and lactic acid assays

Periosteal cells were plated at a density of 1.5 × 10^4^ cells/cm^2^ overnight and subsequently cultured with or without FGL2 recombinant proteins for 48 h. Control wells without periosteal cells were established to serve as blanks. Following the culture period, the media were collected and centrifuged at 800 g for 5 min. The supernatants were preserved for further analysis. Glucose Assay (Beyotime, S0201S) and lactic acid production (Njjcbio, A019–2–1) were assessed following the manufacturer’s instructions.

### Mitotracker

Periosteal cells were plated at a density of 1.5 × 10^4^ cells/cm^2^ overnight and then cultured with or without FGL2 recombinant proteins for 24 h. Cells were stained with MitoTracker Red (Invitrogen, M7512) at 37 °C for 30 min. The cell samples were then fixed in 4% paraformaldehyde for 15 min, rinsed with PBS, and subsequently incubated in a diluted DAPI solution (Beyotime, P0096) for 10 min. After rinsing with PBS, the samples were mounted using an anti-fading mounting medium (Elabscience, E-IR-R119). Images were captured using a fluorescence microscope(Leica, STELLARIS 5).

### Uni-cortical drill-hole tibial model

Drill-hole surgery method was as previously described [21]. All tibial procedures were conducted under sterile conditions. Animals were anesthetized with isoflurane (induction at 3–4%, maintenance at 1.5–2% in oxygen) prior to surgical procedures. An 8–10 mm skin incision was made over the area, and a 0.7 mm hole was drilled through one side of the tibial cortex using a STRONG204 micro electric motor. Following the drilling, the inner tissue layers were first closed using absorbable sutures, followed by closure of the skin using 6/0 non-resorbable surgical sutures. Triple antibiotic ointment was carefully applied to both the surrounding skin and sutures to prevent infection. To alleviate postoperative pain, Buprenex (buprenorphine, 0.05 mg/kg, subcutaneously) was administered immediately after surgery and every 12 h for 48 h.

### Bone sample preparation and μCT analysis

After dissection, femurs and tibias were fixed in 4% paraformaldehyde for 48 h and subsequently stored in 70% ethanol. μCT scans were performed using the NEMO® μCT system (NMC-200; Pingseng Healthcare, Kunshan, China). Scanning was conducted at a voltage of 90 kV and a current of 40 μA. For in vitro bone tissue analysis, three-dimensional (3D) images were reconstructed at a resolution of 4 K × 4 K, with an isotropic voxel size of 2 μm. The following parameters were assessed for the distal femoral trabecular bone beneath the growth plate: bone volume (Tb.BV), total volume (Tb.TV), bone volume fraction (Tb.BV/TV), trabecular thickness (Tb.Th), trabecular number (Tb.N), and trabecular separation (Tb.Sp). The femoral diaphyseal cortical bone was analyzed for bone area (Ct.ar), total area (Tt.ar), and cortical thickness (Ct.Th). For quantification, cross-sectional μCT slices from 0.4 mm to 1.5 mm below the distal femoral growth plate were selected to evaluate trabecular bone parameters, while a 1 mm region at the mid-diaphysis was used for cortical bone assessment. A region of interest (ROI) with a diameter and height of 0.6 mm, centered at the midpoint of the drill-hole site, was defined as the total volume (TV) for quantitative analysis. For in vivo bone tissue analysis, 3D images were reconstructed with a resolution of 1 k x 1 k. All presented 2D and 3D images are representative of the analyzed genotypes.

### Histology

After micro CT scanning, samples were decalcified in 14% ethylenediamine tetraacetic acid (EDTA) for 10 to 14 days under agitation. Samples were embedded in paraffin and sectioned consecutively in 6 μm. Before staining, sections were deparaffinized as follows: Femoral sections were stained with hematoxylin and eosin (H&E). Tibial drill-hole sections were stained with Hematoxylin–Eosin Stain kit (H&E) (Njjcbio, D006), masson-trichrome (Solarbio, G1340), Safranin O, and tartrate-resistant acid phosphatase (TRAP) (Servicebio, G1050).

### Immunofluorescence and confocal imaging

The femurs and tibias were sectioned consecutively in 10 μm at −30℃ using a cryostat machine (Leica). Cryostat sections were blocked with 3% FBS in PBS for 30 min and then incubated in diluted primary Periostin (abcam, ab215199) antibody at 4℃ overnight. After rinsed 3 times with PBS, sections were incubated in diluted secondary antibody for 1 h. Sections were rinsed with PBS for 3 times and subsequently incubated in diluted DAPI solution for 5 min. Sections were rinsed with PBS rinse 5 min and mounted with anti-fluorescence quenching agent (Elabscience, E-IR-R119). Images were visualized with a fluorescence microscope(Leica, STELLARIS 5).

### EdU incorporation assays

A BeyoClick™ EdU Cell Proliferation Kit with Alexa Fluor 488 (Beyotime, C0071S) was used to target proliferating cells. For in vivo EdU labeling, the EdU was dissolved in PBS at a concentration of 2 mg/ml. Intraperitoneally injections of EdU (5 μl/g of body weight) were administered to mice at 18 h, 12 h and 6 h pre-sacrifice for 3 doses. Preparation of frozen sections and incubation of anti-Sca1 (Invitrogen, 14–5981-82) followed by secondary incubation (Alexa Fluor® 546 goat, Invitrogen, cat#A11081) were performed as described above. After samples preparation, EdU click reaction and nuclear counterstaining were performed according to the manufacturer’s instructions. Images of cells or sections were visualized with a ImageXpress Pico automated cell imaging system (Molecular Devices) or the fluorescence microscope, respectively.

### Immunohistochemistry

Paraffin sections were prepared and deparaffinized as described above. The sections were incubated in the citrate buffer (0.0175 M citric Acid + 0.0825 M tri-sodium citrate dihydrate) at 60℃ overnight before incubated with primary antibody against FGL2 (Proteintech, 11827–1-AP, 1:100). Secondary detection were performed using Rabbit 2-step kit (zsbio, PV-6001) and DAB substrate (Solarbio, DA1010) according to the manufacturer’s instructions, followed by the counterstaining with hematoxylin.

### Histomorphometry

For dynamic histomorphometric analysis, mice were subjected to double labeling by intraperitoneal injection of calcein (Sigma, C0875) on day 7 and alizarin red (Sigma, A3882) on day 2 prior to sacrifice. Following tissue harvesting, bones were fixed in 4% paraformaldehyde (PFA) for 48 h at room temperature and subsequently immersed in 30% sucrose for another 48 h for cryoprotection. Samples were sectioned at 10 μm thickness using cryofilm type IIC (Section-Lab, Japan, CFS 105) to preserve bone morphology. Fluorescence imaging was performed using Leica STELLARIS 5, and bone formation parameters including surface per bone surface (MS/BS), mineral apposition rate (MAR), and bone formation rate (BFR) were quantified using BIOQUANT OSTEO software (version 2021 V21.5.60).

### Human specimens

Human periosteum samples were prepared from surgical discard specimens obtained from patient with T2DM undergoing surgery (*n* = 2). The human study was performed in compliance with the official ethical guidelines and protocols approved by the Ethics Committee of the Affiliated Anji People’s Hospital, Hangzhou City University School of Medicine (approval number: P20240228-4). Written informed consent was obtained from the donor prior to the collection.

### Isolation and preparation of periosteal cells for scRNA-seq

All cells were freshly isolated using enzymatic digestion and then processed for single-cell RNA sequencing (scRNA-seq) following cell sorting. Periosteal cells were collected from two groups: non-surgery and 7 days post-drill hole surgery, with each group consisting of pooled samples from 5 db/+ or db/db mice. For the non-surgery group, periosteal cells were isolated following the protocol described above. For the drill hole surgery group, at day 7 post-surgery, the tibias were dissected, ensuring that surrounding muscles were removed while retaining those connected to the periosteum. This step was crucial because complete removal of muscles post-surgery could result in the loss of the periosteum. After isolating the tibias, the same enzymatic digestion protocol was applied to dissociate cells from the entire bone, including the drill hole area. The resulting cell suspension was treated with red blood cell lysis buffer to eliminate red blood cells and subsequently stained with APC-conjugated anti-CD45 antibody (BioLegend, 103112). CD45 negative (CD45^-^) cells were then isolated using fluorescence-activated cell sorting (FACS). The sorted periosteal cells were further processed for single-cell RNA sequencing.

### scRNA-seq analyses

A single-cell library was created using the Chromium Single Cell 3’ Library Kit v3 (10 × Genomics). Library sequencing was carried out on an Illumina NovaSeq PE150 platform. Aligned reads and gene-barcode matrices were generated from FASTQ files, which included Read 1, Read 2, and the i7 index, using the Cell Ranger software package (version 3.0) with default parameters. R (version 4.3.0) and RStudio (version 2023.03.1 + 446) were used for processing of single-cell RNAseq raw data. Seurat (version 4.0.1) was used for further analysis and visualization. Genes expressed in < 3 cells and cells expressing < 200 genes were excluded. Cells expressing 200–9000 genes and expressing < 10% of mitochondrial genes were reserved. To reduce batch effects, the SelectIntegrationFeatures function was performed to identify the top 2000 overlapping variable genes across samples. Based on above genes, the FindIntegrationAnchors function was subsequently performed to identify Integration anchors. Normalization was performed in all data. After scaling and log transformations, principal component analysis (PCA) reduction with the first 20 principal components was performed. To identify clusters, the FindNeighbors and FindClusters functions were performed with 20 dimensions of the PCA and a resolution of 0.5, respectively. Non-linear dimensionality reduction was performed through uniform Manifold Approximation and Projection (UMAP). The DotPlot and FeaturePlot functions were used for gene visualization.

### Gene Ontology (GO) analysis

Seurat (version 4.0.1), ClusterProfiler (version 4.6.2) were used for GO analysis [26]. The entrezIDs of genes were by acquired by org.Hs.eg.db (version 3.16.0, for human) and org.Mm.eg.db (version 3.17.0,for mice). Differentially up-regulated or down-regulated genes of each sample were identified through the FindAllMarkers function of Seurat. The enriched GO terms were present based on biological process.

### Statistical analysis

All statistical analyses were performed using GraphPad Prism (version 9.3.0). Data are presented as mean ± standard deviation (SD) unless otherwise indicated. Normality of data distribution was assessed using the Shapiro–Wilk test. For comparisons between two groups, unpaired two-tailed Student’s t-tests were used when data met assumptions of normality and equal variance. In cases of unequal variance, Welch’s correction was applied. For comparisons among three or more groups, one-way or two-way ANOVA was used, followed by appropriate post hoc tests (Sidak’s multiple comparisons). Variance homogeneity was evaluated using the F-test (for two groups) or the Brown-Forsythe test (for multiple groups). When data did not meet the assumptions of normality, non-parametric tests such as the Mann–Whitney U test were applied. * *P* < 0.05, ** *P* < 0.01,*** *P* < 0.001.

## Results

### Decreased bone mass in type 2 diabetic mice

**Fig. 1:**
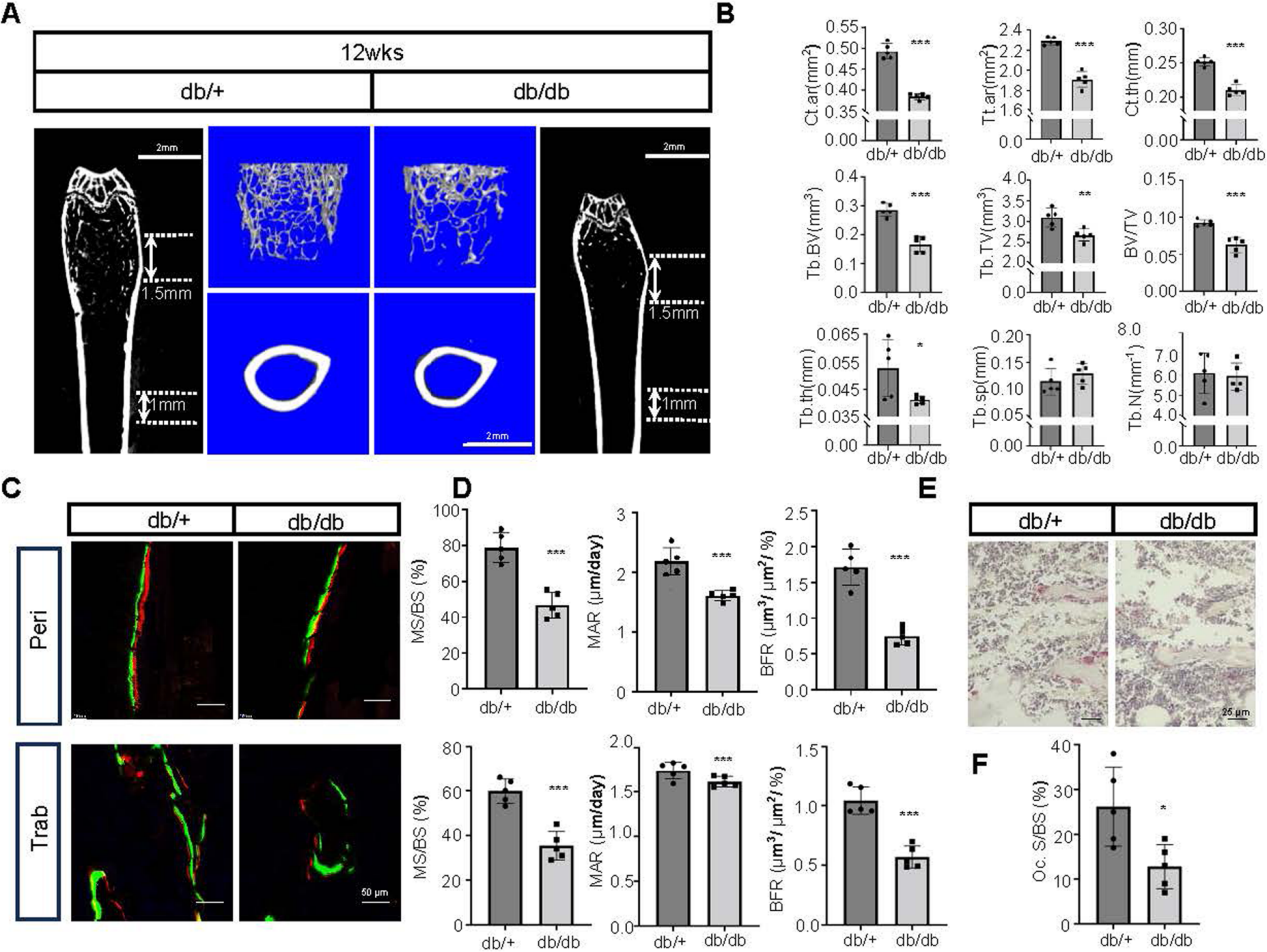
Reduced bone mass in T2DM periosteum. **A** Representative 2D μCT images of the coronal plane in the distal femurs, along with 3D μCT images of 2 mm trabecular bone segments (located 1 mm below the growth plates) and 1 mm cortical bone segments in the mid-diaphysis from db/db and db/+ mice at 12 weeks of age. Scale bar: 2 mm. **B** Quantitative analysis of cortical area (Ct.ar), total tissue area (Tt.ar), and cortical thickness (Ct.th) for the 1 mm cortical bone segments in the mid-diaphysis, and trabecular bone volume (Tb.BV), trabecular area (Tb.ar), trabecular thickness (Tb.th), bone volume fraction (BV/TV), trabecular separation (Tb.sp), and trabecular number (Tb.N) for the 2 mm trabecular bone segments in the distal femurs from db/db and db/+ mice at 12 weeks of age. **C** and **D** Representative images and quantification of double labeling in periosteal and trabecular regions in db/+ and db/db mice. Scale bars: 50 μm. **E** and **F** Representative images and quantification of TRAP staining in trabecular bone. Scale bars: 25 μm. Statistical significance was assessed using a two-tailed Student’s t-test. Data are presented as mean ± SD (*n* = 5)

Recent studies have demonstrated a decrease in bone mass in youth-onset type 2 diabetic models [20]. In our study, we utilized db/db mice as a model for type 2 diabetes. Compared to the control group, these mice exhibited higher body weight and hyperglycemia starting at 8 weeks of age (Figure S1A). We first employed in vivo μCT to monitor changes in bone mass over time. By 12 weeks of age, the in vivo μCT analysis revealed a decrease in femoral cortical bone thickness in db/db mice compared to controls (Figure S1D, E). Mice were sacrificed at 12 weeks, and femurs were collected for further analysis. Stereomicroscope and μCT analyses demonstrated significantly shorter femur lengths in db/db mice compared to controls (Figure S1B, C). Further μCT analysis of the femurs indicated a significant decrease in bone volume in db/db mice (Fig. 1A and Figure S1F). Analysis of the trabecular bone parameters 1–3 mm below the femoral growth plate showed significant reductions in trabecular bone volume (Tb.BV), total area (Tt.Ar), trabecular thickness (Tb.Th), and bone volume fraction (BV/TV) in db/db mice, while trabecular spacing (Tb.Sp) showed no significant differences. Analysis of the mid-femoral shaft (1 mm from the center) revealed significant reductions in cortical bone parameters, including cortical area (Ct.Ar), total area (Tt.Ar), and cortical thickness (Ct.Th) in db/db mice (Fig. 1B). In summary, by 12 weeks of age, db/db mice exhibited reduced femoral cortical bone thickness and decreased trabecular bone volume. To further determine whether alterations in bone formation and resorption contribute to the reduced bone mass observed in db/db mice, we performed dynamic histomorphometric analysis, which revealed significant decreases in both MS/BS, MAR and BFR in the trabecular and periosteal regions (Fig. 1C and D). In addition, histomorphometric assessment using TRAP staining showed a marked reduction in osteoclast numbers in 12-week-old db/db mice (Fig. 1E and F). These results demonstrate that the low bone mass phenotype observed in this early-onset T2D model is primarily attributable to impaired bone formation.

### Altered periosteal cell composition in type 2 diabetic mice

**Fig. 2:**
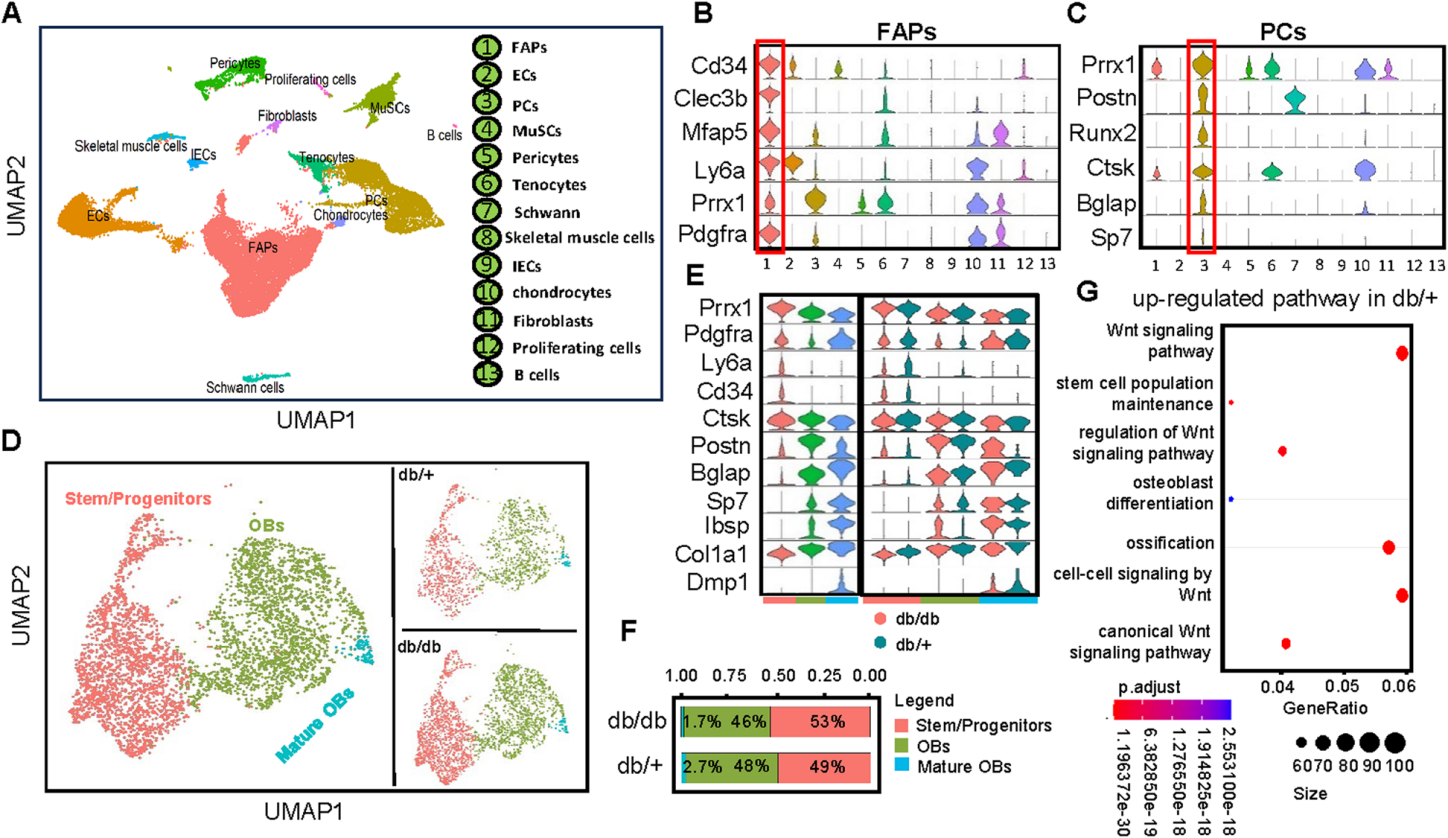
Features of periosteal cells in wild-type and type 2 diabetic mice. **A** sc-RNA seq of femoral and tibial periosteal cells (PCs) from db/+ mice at 12 weeks old. Cells were pooled from 5 samples for each dataset. UMAP visualization shows 13 clusters. FAPs = fibro-adipogenic progenitors; ECs = endothelial cells; lECs = lymphatic endothelial cells; MuSCs = muscle satellite cells; PCs = periosteal cells. **B** and **C** Violin plots showing the expression levels of representative markers for FAPs and PCs. **D** Re-clustering of PCs with UMAP projections displayed for both integrated and individual datasets from db/db and db/+ mice, identifying 3 subpopulations. **E** Vlnplots illustrating the expression levels of representative marker genes across the 3 subpopulations of PCs from db/db and db/+ mice. **F** Percentages of different subpopulations in PCs from db/db and db/+ mice. **G** Enriched osteogenesis-related Gene Ontology (GO) terms for upregulated genes in PCs from db/+ mice. Dot size represents the number of enriched genes, and the color bar reflects the adjusted *P* value. *P < 0.05, **P < 0.01, ***P < 0.001. UMAP = uniform manifold approximation and projection; GO = gene ontology; db/db, the db/db mice group; db/+, the wild-type mice group

The reduction in bone mass, especially cortical bone thickness, in db/db mice may be related to changes in the periosteum. To further elucidate the cellular composition and heterogeneity of the periosteum in db/db mice, we isolated periosteal cells from the long bones of 12-week-old db/+ and db/db mice. These cells were isolated using enzyme and subsequently sorted for CD45^−^ cells (Figure S2A). The sorted cells were then subjected to 10 × scRNA-seq and integrated with scRNA-seq data from db/+ mice. After sequencing, 13,845 cells from the db/+ dataset and 12,670 cells from the db/db dataset were obtained. The two datasets were integrated, resulting in the identification of 13 distinct clusters (Fig. 2A). Our enzymatic digestion method was designed to encapsulate both epiphyseal ends, but agarose gel detachment may have occurred during the process. To maintain the integrity of the periosteum, we also preserved the muscles attached to it. Consequently, our cell population contains subsets of both chondrocytes and muscle cells. These clusters included fibro-adipogenic progenitors (FAPs) by expression of platelet-derived growth factor receptor-α (*Pdgfra*), periosteal osteogenic cells by expression of Runt-related transcription factor 2 (*Runx2*), endothelial cells by expression of endomucin (*Emcn*), muscle satellite cells by expression of Paired Box 7 (*Pax7*), pericytes by expression of actin alpha 2 (*Acta2*) skeletal muscle cells by expression of actin alpha 1 (*Acta1*), chondrocytes by expression of Collagen Type II Alpha 1 (*Col2a1*), Schwann cells by expression of Proteolipid Protein 1 (*Plp1*), lymphatic endothelial cells by expression of Lymphatic Vessel Endothelial Hyaluronan Receptor 1 (*Lyve1*), immune cells by expression of Immunoglobulin Heavy Constant Mu (*Ighm*), tenocytes by expression of Scleraxis (Scx), and cycling cells by expression of Marker of Proliferation Ki-67 (*Mki67*) (Fig. 2A, Figure S2 B-I and Table S2).

The FAPs cluster exhibits high expression of Mesenchymal Stem Cells (MSC) marker genes Ly6a, Prrx1, and Pdgfra [27] (Fig. 2B). Our primary focus, the periosteal cells cluster, shows high expression of Postn, Ctsk, Runx2, and Bglap (Fig. 2C). We further analyzed the periosteal osteogenic lineage cells, which contribute to bone formation. We identified Pdgfra^+^/Ly6a^+^ fibrous layer and Postn^+^/Osx^+^ cambium layer periosteal cells [9], as well as Dmp1^+^ mature osteoblasts in the periosteal osteogenic lineage (Fig. 2D and E). To assess the heterogeneity of the periosteum in db/db versus db/+ mice, we visualized the proportion of osteogenic lineage cell types and the expression of marker genes. Expectedly, the proportion of osteoblasts was lower in db/db mice than in db/+ mice (Fig. 2F). GO pathway enrichment analysis showed significant downregulation of the Wnt pathway in db/db mice (Fig. 2G). In summary, the heterogeneity of the periosteal osteogenic lineage in db/db mice is characterized by the downregulation of osteogenesis-related pathways, as well as a reduction in the proportion of osteoblasts.

### Periosteal cell composition in type 2 diabetic patients

**Fig. 3:**
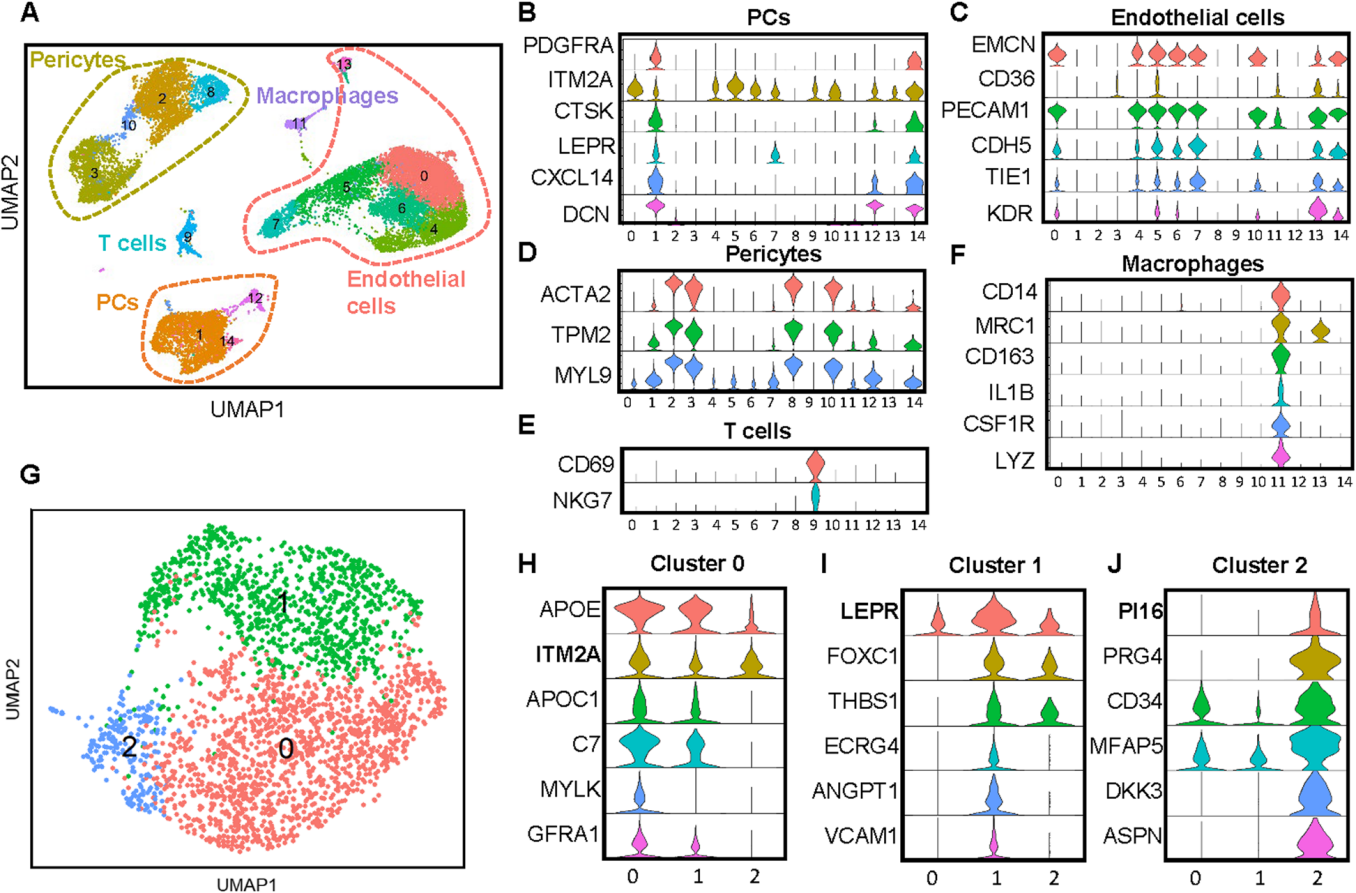
scRNA-seq analysis reveals distinct cellular populations in the periosteum of T2DM patients. **A** UMAP visualization of unsupervised clustering of periosteal cells, revealing distinct cell populations within the T2DM human periosteum. **B-F** Vlnplots illustrating the expression levels of selected marker genes across different cell populations. **G** UMAP visualization of reclustered periosteal cells, showing the diversity of cell types in the dataset. **H-J** Vlnplots showing the expression of specific genes across cluster 0, 1 and 2. PCs = periosteal cells

To investigate further Periosteal cell features in T2DM, we used the same flow cytometry method to isolate periosteal cells from T2DM human subjects, selecting only CD45^−^ cells, and performed scRNA-seq. In the UMAP analysis of the human periosteum, a total of 19,279 cells were sequenced. Currently, no single-cell atlas of the periosteum in adults or elderly individuals exists. In this study, we identified periosteal cells based on previously reported markers, including PDGFRA [28], CTSK [29], and ITM2A [30]. Among these, we identified clusters of EMCN^+^ endothelial cells (clusters 0, 4, 5, 6, 7, 13), ACTA2^+^ pericytes (clusters 2, 3, 8, 10), NKG7^+^ T cells (cluster 9), LYZ^+^ macrophages (cluster 11), and PDGFRA^+^ cell clusters of interest (clusters 1, 12, 14) (Fig. 3A-F and Supplemental Table 3). We further reclustered the PDGFRA cell population, which also shows high expression of LEPR, CXCL14, and DCN (Fig. 3B), and identified three distinct clusters (Fig. 3G). Previous studies have identified Pi16 as a potential periosteum progenitor marker in mice [31], and in our dataset, Cluster 2 exhibited high expression of PI16. However, Cluster 2 also expressed markers associated with fibro-adipogenic progenitors (FAPs), including CD34 and MFAP5 (Fig. 3J). In contrast, Cluster 0 displayed high expression of ITM2A, a marker reported to identify periosteal cells in mice [30] (Fig. 3H). Cluster 1 exhibited elevated expression of LEPR, which has been shown in previous studies to target periosteal cells in Lepr-cre mouse models [7] (Fig. 3I). Based on the expression patterns observed in the violin plots, both human and mouse periosteum express PDGFRA, CTSK, and ITM2A.

### Delayed bone healing and impaired periosteal response in type 2 diabetic mice

To better understand the role of the periosteum in intramembranous bone healing under T2DM conditions, we utilized the drill hole injury model to minimize the impact of muscle and bone marrow cells on the healing process, with a primary focus on the contribution of periosteal cells. Since there was no systematic observation of periosteal changes during intramembranous bone healing, we first conducted drill hole experiments on wild-type (db/+) mice to monitor the periosteum’s response from day 0 to day 21 post-surgery (Figure S3A-F). By day 5 post-surgery, the periosteum had expanded and migrated towards the defect site, with early woven bone formation observed within the defect and the bone marrow cavity (Figure S3C). By day 7, significant periosteal cell proliferation covered the drill hole area, accompanied by extensive woven bone formation at the site and within the bone marrow cavity (Figure S3D). New bone formation peaked between days 7 and 14 post-surgery (Figure S3D and E). The periosteum was thickest on day 7 post-surgery but decreased noticeably by day 14, as new lamellar bone began to fill the defect, gradually restoring the bone structure. By day 21, the lamellar bone had nearly completely covered the defect, though the cortical bone thickness remained slightly reduced compared to preoperative levels (Figure S3F).

**Fig. 4:**
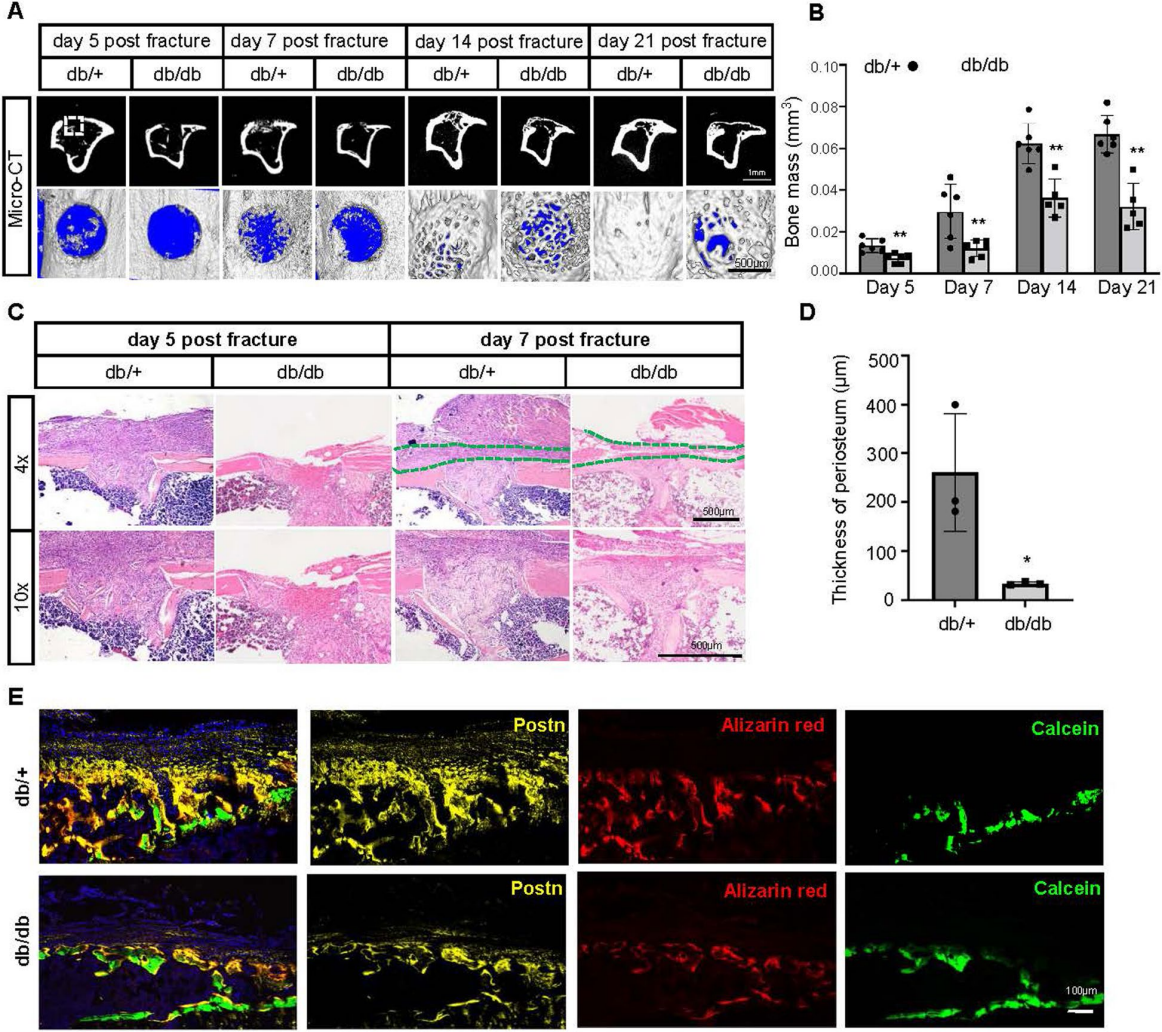
Delayed fracture healing and reduced proliferating cells in type 2 diabetes. **A** Representative 2D μCT images of horizontal plane and 3D μCT images of the tibias from db/db and db/+ mice 5,7, 14 and 21 days after fracture. Scale bar: 1 mm and 500 μm. **B** Quantification of newly formed bone mass in the fractured sites (outlined in white) of tibias from db/db and db/+ mice 5, 7, 14 and 21 days after fracture. Statistical significance was assessed using two-way ANOVA. Data are presented as mean ± SD (*n* = 6). **C** Haematoxylin and eosin (H&E) staining of tibias from db/db and db/+ mice 5 or 7 days after fracture. Green lines outline the periosteum. Scale bar: 500 μm. **D** Quantification of periosteal thickness in db/+ and db/db mice at day 7 post-fracture. Data are presented as mean ± SD. **E** Representative images of double labeling in the surgery region, showing immunofluorescence for periostin (Postn, yellow) and mineral labeling with alizarin red (red) and calcein (green) in db/+ and db/db mice. Scale bars: 100 μm.

Subsequent experiments primarily focused on changes observed on days 5, 7, 14 and 21 post-surgery. We conducted drill hole surgeries on both db/+ and db/db mice. CT scans revealed a significant reduction in new bone formation in db/db mice at both time points compared to db/+ mice (Fig. 4A, B; Figure S4A, B). Additionally, HE staining showed a considerable decrease in periosteal thickness in the T2DM model, particularly notable at the critical 7-day mark post-surgery, with a reduced number of periosteal repair cells (Fig. 4C, D). To further clarify the alterations in bone formation and resorption under diabetic fracture conditions, dynamic histomorphometric analysis showed a significant reduction in mineral apposition rate (MAR) in db/db mice during the bone healing process (Figure S4C, D). Immunostaining for periostin (POSTN) also revealed a notable decrease in periosteal thickness (Fig. 4E). In parallel, TRAP staining indicated reduced osteoclastic activity in db/db mice, suggesting a decrease in bone resorption as well (Figure S4E, F). Our findings highlight that periosteal-mediated bone repair is significantly compromised in db/db mice due to reduced bone formation.

### scRNA-seq reveals impaired proliferation of periosteal cells in T2DM leading to delayed bone healing

**Fig. 5:**
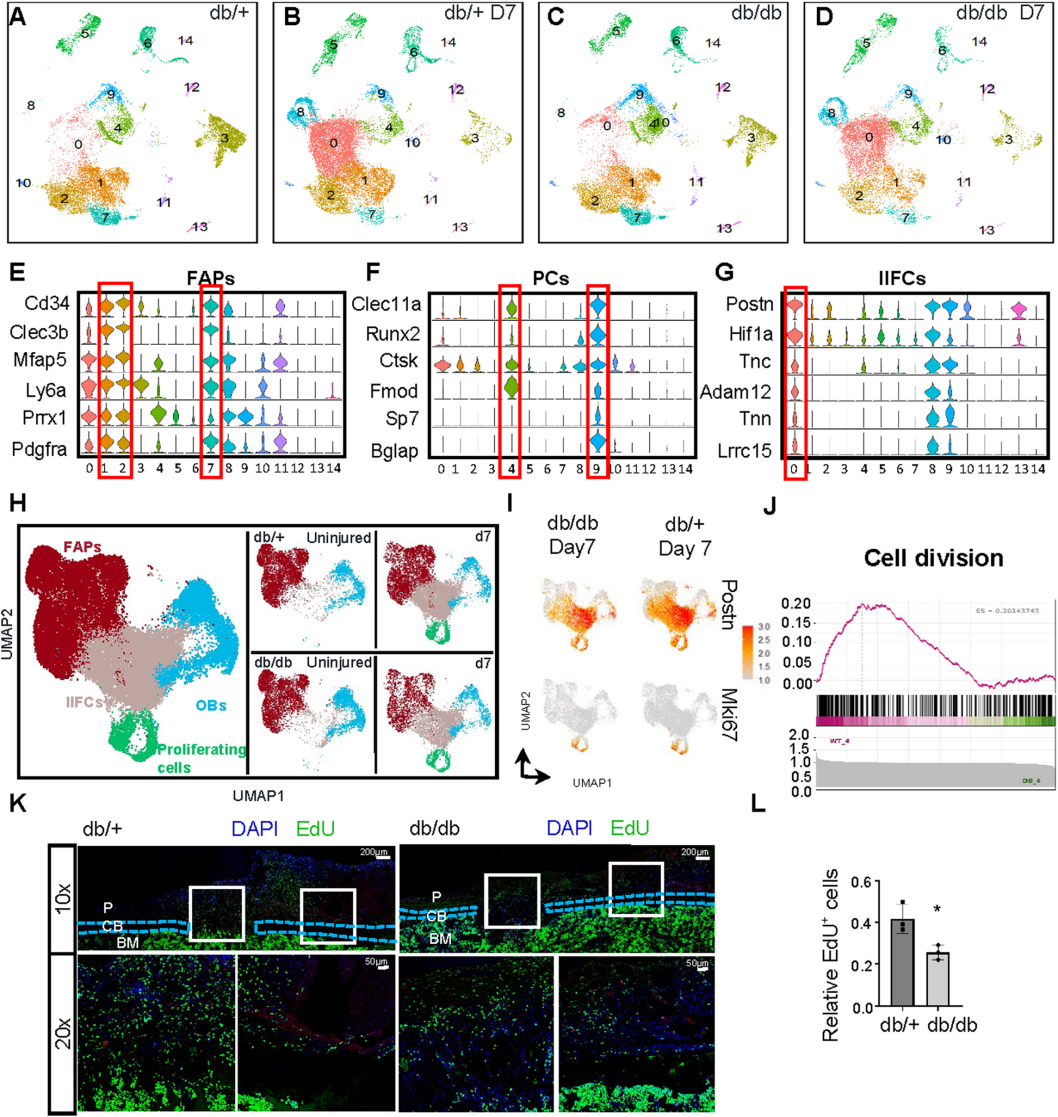
Integrated analysis of periosteal cells in db/db and db/+ mice pre- and post-fracture.** A-D** UMAP visualization of unsupervised clustering of cells from normal periosteum and injured periosteum 7 days post-fracture from db/db and db/+ mice, following integrated analysis. UMAP visualization displays 15 clusters. Cells were pooled from 5 samples for each dataset. **E–G** Violin plots showing the expression levels of representative markers for FAPs, PCs and IIFCs. **H** Re-clusterization of PCs, FAPs, IIFCs and proliferating cells. UMAP projects were presented in both integrated and individual dataset from db/db and db/+ mice, uninjured or fractured. 5 subpopulations were defined. FAPs = fibro-adipogenic progenitors; PCs = periosteal cells; IIFCs = injured-induced fibrogenic cells. **I** Feature plots depicting the expression levels of the representative marker genes Postn and Mki67 across five subpopulations in the db/+ and db/db injury periosteum dataset. **J** GSEA analysis of the proliferating cell cluster shows a significant upregulation of cell division in db/+ vs. db/db. **K** Top: Representative confocal images of tibias from db/db and db/+ mice 7 days after fracture. Scale bar: 200 μm. Bottom: Enlarged view of the outlined regions in white from the top panel. Scale bar: 50 μm. The green, red, and blue channels reflect EdU, Ly6a, and DAPI staining, respectively. **L** The ratio of EdU^+^ cells to DAPI^+^ cells in the fractured sites of tibias from db/db and db/+ mice 7 days after fracture. Statistical significance was determined using a two-tailed Student’s t-test. Data are presented as mean ± SD (*n* = 3). *P < 0.05, **P < 0.01. P = periosteum; CB = cortical bone; BM = bone marrow

To elucidate the mechanisms underlying delayed intramembranous bone healing in type 2 diabetes mellitus (T2DM), we conducted single-cell RNA sequencing (scRNA-seq) on freshly isolated periosteal cells from db/+ and db/db mice 7 days after drill hole surgery, yielding 16,937 and 10,913 cells, respectively. Integration of the scRNA-seq datasets from db/+ and db/db mice, both before and after surgery, identified 25 distinct cell clusters (Fig. 5A-D), including FAPs (Clusters 1, 2, and 7), pericyte clusters (Clusters 4 and 9), injury-induced fibrogenic cells (Cluster 0) [31], proliferating cells (Clusters 8 and 14), muscle stem cells (MuSCs; Cluster 6), pericytes (Cluster 5), Schwann cells (Cluster 13), tenocytes (Cluster 4), endothelial cells (Clusters 3 and 12), and chondrocytes (Cluster 10) (Fig. 5E-G, Figure Supplemental 5A-G and Supplemental Table 4). Previous studies have demonstrated that muscle also plays a role in fracture healing, so we included the FAPs cluster in our subsequent analysis [27]. Further in-depth analysis was then performed on FAPs, osteoblasts, and proliferating cells (Fig. 4H). Notably, both db/+ and db/db mice exhibited two distinct cell clusters 7 days post-surgery: an injury-induced fibroblast cluster (IIFC) and a proliferating cell cluster (Fig. 5H). The IIFC cluster showed fibrogenic features, expressing Postn and mesenchymal markers such as Ly6a, Clec3b, and CD34. It also expressed the osteoblast progenitor marker Runx2, suggesting potential osteogenic commitment. The proliferating cluster was defined by high levels of cell cycle–related genes including Mki67, and shared a transcriptional profile similar to the IIFC (Fig. 5I and Figure S5 H). To further explore differences between db/+ and db/db mice, we analyzed gene expression within each cluster post-surgery. However, no major differences were observed in the expression of these key marker genes between the two genotypes (Figure S5I).

Given our previous hematoxylin and eosin (H&E) staining results showing reduced cellularity around the drill hole in db/db mice, we focused specifically on the proliferating cell cluster. Gene set enrichment analysis (GSEA) revealed significant downregulation of cell division pathways in db/db mice (Fig. 5J). To validate this finding, we performed EdU labeling of proliferating cells around the drill hole and confirmed the reduction in cell proliferation through immunofluorescence staining (Fig. 5K and L). These findings further substantiate the impaired periosteal cell proliferation in db/db mice and highlight the emergence of injury-induced fibrogenic and proliferating cell clusters as key features of the periosteal response to injury, offering insights into the cellular basis of delayed bone healing in T2DM.

Due to the differences in surgical methods for bone fractures, bone repair can occur through either intramembranous ossification or endochondral ossification. Drill hole surgery is an example of intramembranous ossification [21]. To further elucidate the mechanisms of bone healing, we integrated our single-cell data with other published datasets from various bone fracture surgeries, focusing on cells from the periosteum and bone callus. We used data from Lutian Yao et al., who performed closed transverse fractures on Col2-Cre;Ai9 mice and sorted periosteum Td^+^ cells from day 0 before fracture, day 5 after fracture, and day 10 after fracture [32]. We integrated a total of seven datasets and generated a merged UMAP, identifying 26 clusters (Figure S6A). These clusters included endothelial cells (Emcn^+^), pericytes (Acta2^+^), muscle stem cells (Pax7^+^), Schwann cells (Plp1^+^), lymphatic endothelial cells (Lyve1^+^), immune cells (Ighm^+^), proliferating cells (Mki67^+^), FAPs (Pdgfra^+^), osteoblasts (Sp7^+^), and chondrocytes (Col2a1^+^) (Figure S6B). We further reclustered the clusters of interest, specifically clusters 0, 1, 3, 4, 6, 8, 9, 11, 14, 17, and 22, and generated a reclustered UMAP (Figure S7A-G), identifying FAPs, periosteal cells (PCs), IIFCs, chondrocytes (CHs), and proliferating cells (Mki67^+^) (Figure S7H). In our intramembranous ossification model, the presence of CHs is likely an artifact caused by agarose detachment during enzymatic processing. Feature plots further revealed that in the tibia fracture model, Acan expression peaks at day 10 in tibia fracture model (Figure S7I). However, injury-induced fibrogenic features were observed in both intramembranous and endochondral ossification processes. IIFCs were found to highly express Postn and Acta2 in both models (Figure S7J, K). The Mki67^+^ cluster and IIFCs cluster display highly similar gene expression profiles, with both expressing fibrogenic markers Postn and Tpm2, the osteogenic marker Runx2, cartilage markers Acan and Sox9, and FAPs markers Pdgfra, Dpt, and Cd34 (Figure S6H). In summary, injury-induced fibrogenic cells were identified as key contributors in both intramembranous and endochondral ossification processes, highlighting their importance in the periosteal response to injury.

### Increased expression of FGL2 in drill hole sites of T2DM mice

**Fig. 6:**
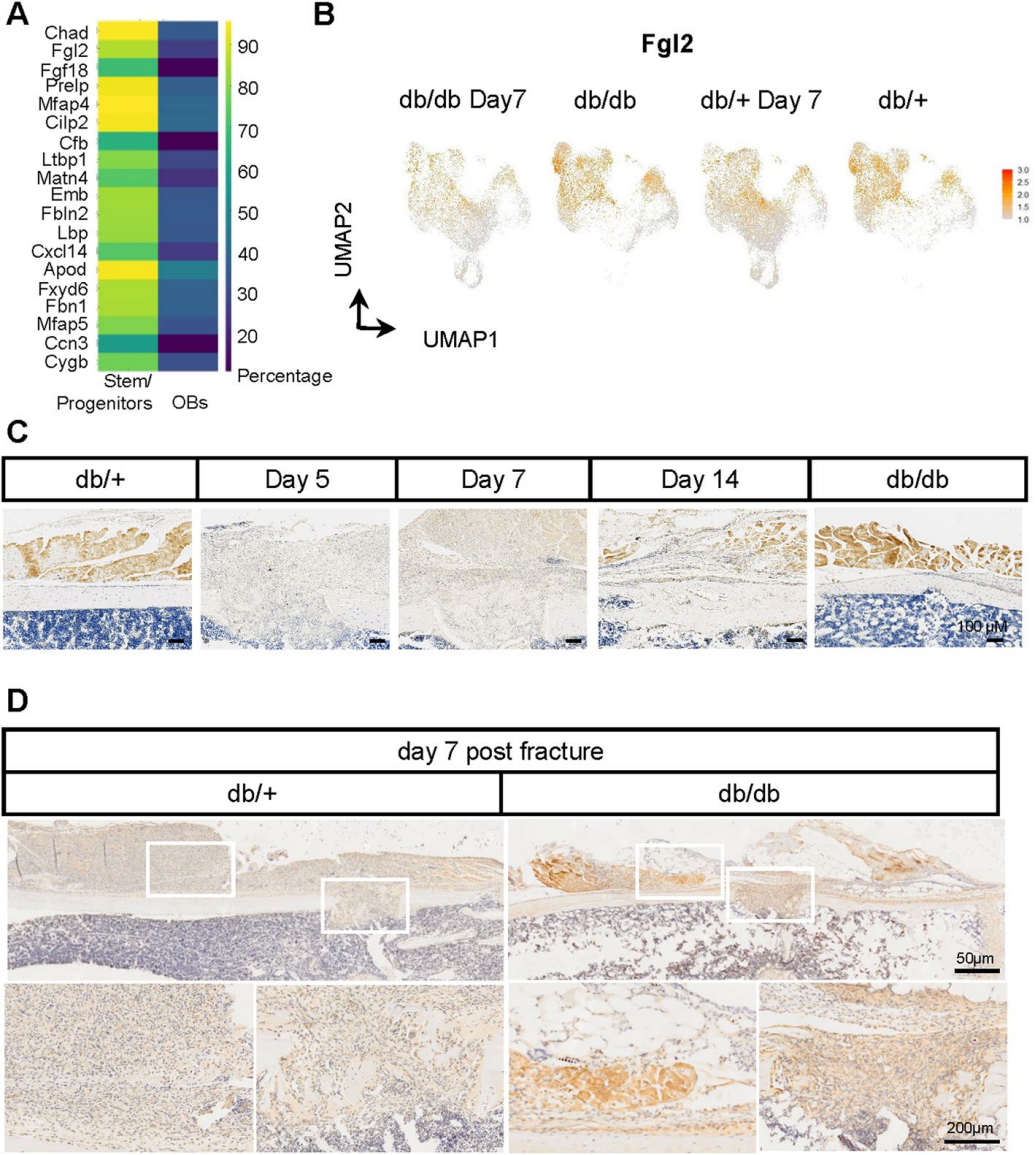
Analysis of gene expression and histological changes in periosteal cells post-fracture. **A** Heatmap displaying the expression of top marker genes across different periosteal cell populations, including stem/progenitors and OBs. **B** Feature plots illustrating the expression of *Fgl2* in periosteal cells under various conditions, including db/db and db/+ mice 7 days post-fracture, as well as in db/+ and db/db mice under normal conditions. The color intensity represents the level of *Fgl2* expression. **C** Histological sections showing the temporal expression pattern of FGL2 during bone healing in db/+ mice at baseline (non-surgery), and at days 5, 7, and 14 post-fracture, as well as in db/db mice. Scale bar: 100 µm.** D** Comparative histological analysis of FGL2 between db/+ and db/db mice at 7 days post-fracture. Scale bars: 50 µm and 200 µm, as shown

During differential gene analysis of the FAPs and osteogenic cell clusters, we identified a highly expressed secreted protein, Fibrinogen-like protein 2 (FGL2) (Fig. 6A). *Fgl2* was expressed in both FAPs and periosteal osteogenic cells (Fig. 6B). Immunohistochemistry (IHC) confirmed its expression in both muscle and periosteal cells in the normal group, with significantly higher expression in muscle compared to the periosteum. Following the drill hole surgery, we observed uniform expression of FGL2 in both the muscle and the thickened periosteum (Fig. 6C). Additionally, we assessed FGL2 expression levels post-surgery in db/+ and db/db mice, finding a significant increase in both the surrounding connective tissue and periosteum in the db/db group (Fig. 6D). We hypothesize that elevated FGL2 levels may influence the proliferation of periosteum and injury-induced fibroblast cells. These findings indicate that the elevated expression of FGL2 may play a pivotal role in regulating the proliferative dynamics of periosteal and injury-induced fibroblast populations, potentially contributing to dysregulated tissue repair processes in diabetic conditions.

### FGL2-mediated activation of the mTOR signaling pathway promotes periosteal cell proliferation

**Fig. 7:**
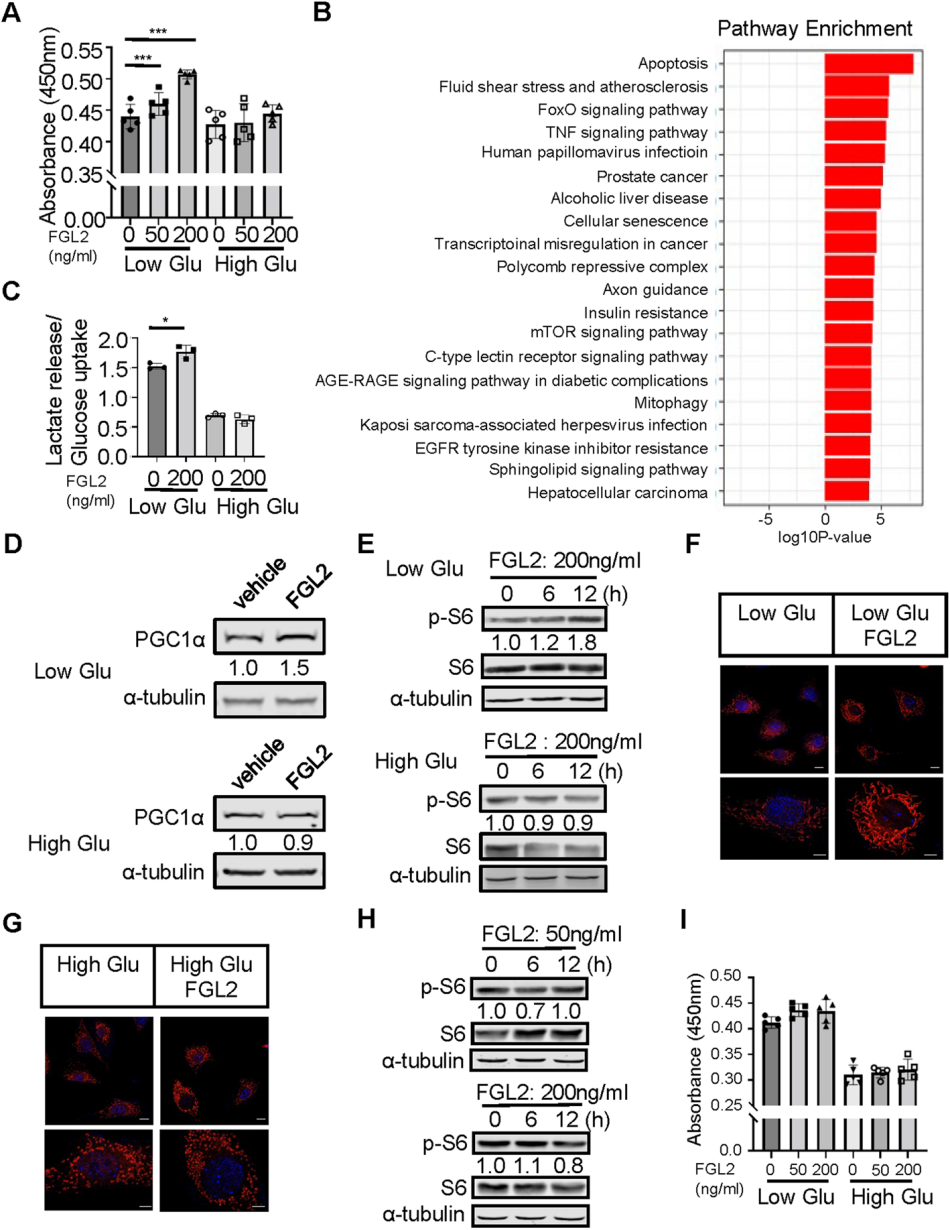
Effects of FGL2 on periosteal cells function under normal and glucose-rich conditions. **A** Relative cell proliferation of Periosteal cells under normal and high glucose conditions after 72 h of treatment with varying concentrations of FGL2 recombinant proteins. Statistical significance was assessed using one-way ANOVA with Sidak’s multiple comparison test. Data are presented as mean ± SD. **B** Top 20 upregulated KEGG pathways identified in scRNA-seq periosteal cell clusters, comparing db/+ post-fracture versus db/db post-fracture conditions. **C** Ratio of lactate release to glucose uptake in periosteal cells under normal and high glucose conditions after 48 h of treatment with 200 ng/mL FGL2 recombinant proteins. Statistical significance was determined using a two-tailed Student’s t-test. Data are presented as mean ± SD. **D** Protein levels of PGC1 in periosteal cells under normal and high glucose conditions after 24 h of treatment with 200 ng/mL FGL2 recombinant proteins. **E** Protein levels of phospho-S6 and S6 in periosteal cells under normal and high glucose conditions after 6 and 12 h of treatment with 200 ng/mL FGL2 recombinant proteins.** F** and **G** Confocal images of MitoTracker staining in periosteal cells under normal and high glucose conditions after 24 h of treatment with 200 ng/mL FGL2 recombinant proteins. Channels show MitoTracker (red), and DAPI (blue). Scale bar: 10 μm and 5 μm. **H** Protein levels of phospho-S6 and S6 in *Raptor* KO osteoblasts after 6 and 12 h of treatment with 50 ng/mL and 200 ng/mL FGL2 recombinant proteins. **I** Relative cell proliferation of Raptor KO periosteal cells under normal and high glucose conditions after 72 h of treatment with varying concentrations of FGL2 recombinant proteins. Statistical significance was assessed using one-way ANOVA with Sidak’s multiple comparison test. Data are presented as mean ± SD. *P < 0.05, **P < 0.01, ***P < 0.001

To elucidate the effect of the secreted protein FGL2 on periosteal cells, we isolated these cells in vitro and assessed their proliferative response. The isolated periosteal cells were cultured in normal medium (1 g/L glucose) and high-glucose medium (4.5 g/L glucose). Recombinant FGL2 protein (50, 200 ng/mL) was administered, and cell proliferation was observed at 48 and 72 h post-administration (Fig. 7A and Figure S8A). Under normal glucose conditions, FGL2 significantly promoted periosteal cell (PC) proliferation, whereas no proliferative effect was observed under high-glucose conditions.

We further analyzed scRNA-seq-based signaling pathway changes in periosteal cell clusters from db/+ and db/db mice after fracture. Among the enriched pathways, apoptosis emerged as one of the top regulated pathways in our KEGG enrichment analysis. To investigate the mechanisms underlying impaired periosteal cell proliferation, we also examined the mTOR signaling pathway, which is closely associated with cell proliferation and glucose metabolism, and found that it was downregulated in the db/db group (Fig. 7B). Under normal glucose conditions, FGL2 promoted glycolytic metabolism, enhancing the conversion of glucose to lactate. However, under high-glucose conditions, the conversion of glucose to lactate was significantly reduced, and the effect of FGL2 was diminished (Fig. 7C). Additionally, we observed that FGL2 upregulated the key mitochondrial biogenesis factor PGC-1α protein expression (Fig. 7D) and p-S6 expression (Fig. 7E). Using MitoTracker immunofluorescence, we further examined mitochondrial morphology and fluorescence intensity. Under normal culture conditions, the FGL2-treated group exhibited mitochondria with high fluorescence intensity and filamentous morphology (Fig. 7F). In contrast, under high-glucose culture conditions, the mitochondria appeared with punctate morphology (Fig. 7G).

To further elucidate the role of the mTOR signaling pathway in FGL2-induced periosteal cell proliferation, we used CRISPR-Cas9 to knock out Raptor, a key protein in the mTOR pathway. qPCR analysis showed an approximately 50% reduction in Raptor mRNA levels, while Western blot analysis demonstrated a near-complete loss of Raptor protein expression, confirming efficient knockout at the protein level (Figure S8B, C). Under normal culture conditions, Raptor-knocked out periosteal cells showed no change in p-S6 levels in response to FGL2 stimulation. Similarly, there was no effect under high-glucose conditions (Fig. 7H). Proliferation assays further confirmed that the proliferative effect of FGL2 on periosteal cells was abolished following Raptor knockout (Fig. 7I). These findings indicate that FGL2 facilitates periosteal cell proliferation via the mTOR signaling pathway under normoglycemic conditions, while hyperglycemia significantly impairs this proliferative effect. This disruption likely results from altered mitochondrial dynamics and metabolic dysregulation, potentially contributing to compromised intramembranous bone healing in diabetic environments.

## Discussion

We depicted the periosteal single-cell atlas of normal and T2DM mice before and after intramembranous bone healing, with a particular focus on the changes in glucose metabolism-related signaling pathways. Delayed fracture healing has been well-documented in both clinical settings and animal models of T2DM. Although the periosteum has been studied in fracture models, earlier research faced technical challenges in reliably isolating pure periosteal cells due to contamination from adjacent bone marrow and muscle tissues. Recent advances in single-cell transcriptomics now enable more precise identification based on molecular signatures. In this study, using the drill hole surgical model, we collected the entire periosteal cell population post-surgery without restricting our analysis to specific cell lineages through Cre lineage tracing, providing a comprehensive and unbiased cellular atlas. Additionally, by integrating our data with datasets from Col2-cre; Ai9 mouse models, we further substantiated the critical role of injury-induced fibrogenic cells in both intramembranous and endochondral ossification processes during fracture healing. Finally, we identified the secreted protein FGL2 as a key regulator of mitochondrial biogenesis, driving periosteal cell proliferation. Importantly, under hyperglycemic conditions, the proliferative effect of FGL2 on periosteal cells was abrogated, highlighting its diminished role in the context of T2DM-related impaired healing.

Both high-fat diet-induced T2DM models and db/db mice exhibit delayed fracture healing. Given the previously debated skeletal phenotype of db/db mice, we first confirmed that these mice have significantly reduced bone mass using micro-CT analysis. We then further demonstrated that db/db mice exhibit impaired healing of bone defects, consistent with findings from previous studies. While previous reports have indicated that T2DM inhibits the differentiation of skeletal stem cells into osteoblasts and involves factors such as the sympathetic nervous system, macrophage-mediated bone regulation, and matrix metalloproteases [33–36], the important role of the periosteum in fracture repair has often been overlooked. Although previous literature has indicated a reduced proliferative capacity of periosteal cells during fracture healing in T2DM [37], the study primarily used flow cytometry to isolate lin^−^Sca1^+^CD105^+^ periosteal mesenchymal progenitors. This method does not clearly define the specific cell populations involved in fracture repair and is susceptible to contamination by bone marrow cells in the isolated periosteal cell populations post-fracture. We utilized a drill hole model, which causes minimal damage to the muscle and bone marrow cavity, focusing on periosteum. Our scRNA-seq results thus offer a more detailed and comprehensive insight into the role of the periosteum in delayed intramembranous bone healing under T2DM conditions.

Recent research using Col2-Cre;Ai9 for sc-RNA seq to examine cell population changes at 5 and 10 days post-tibia fracture has reported a significant increase in proliferative progenitor cells (PPCs) [32]. We similarly identified PPCs during intramembranous ossification, with this cluster (IIFC) showing a significant increase post-surgery. However, unlike the cell populations observed following tibia fracture, the proportion of the chondrocyte cluster did not exhibit a notable increase in our drill hole surgery datasets. Other studies employing Pdgfra-CreER and Osx-Dre mice have highlighted the crucial role of the outer-layer periosteum (Cd34^+^Dpt^+^) in fracture repair [9]. Our findings further confirm the crucial role of the outer-layer periosteum (Cd34^+^Dpt^+^) in the process of intramembranous ossification. Therefore, our findings confirm that the proliferation of fibroblasts is a key intermediate process in intramembranous bone healing, regardless of whether the repair occurs through intramembranous or endochondral ossification. Notably, Prx1-Cre targets not only periosteal cells but also muscle-resident fibro-adipogenic progenitors (FAPs), which have been shown to contribute to repair [27]. In addition, Lepr-Cre-based lineage tracing in drill-hole models has highlighted the contribution of bone marrow stromal cells [8]. As our study is focused primarily on the periosteum, we acknowledge that further work is needed to clarify the roles of muscle-derived and bone marrow stromal populations in fracture repair, particularly in the context of T2DM.

Our previous research showed that aerobic glycolysis serves as the primary energy source for osteoblast differentiation, and that enhancing glycolysis can alleviate the osteoporotic phenotype in Akita mice with T1DM-induced osteoporosis [16, 19]. In our current experiments, we observed a significant downregulation of energy metabolism-related signaling pathways, including mTOR and MAPK, in osteoblast subpopulations following fracture in db/db mice. Differential gene analysis has identified that the secreted protein FGL2 is highly expressed in periosteal osteogenic cells, as well as in FAPs. Additionally, when the physical barrier between bone compartments is compromised, the healing response in one tissue may influence the adjacent tissue through the migration of cells and diffusion of growth factors. In the intact periosteum, FGL2 is expressed within the periosteum and is highly expressed in the connective tissue. However, following injury, FGL2 spreads into the damaged soft tissue and fracture site. By 14 days post-injury, FGL2 expression returns to its normal levels. Given its established role in regulating inflammation and extracellular matrix remodeling in other tissues, FGL2 may act as a modulator of bone healing microenvironment, facilitating early repair but being downregulated once the inflammatory phase resolves. Our experiments also found that treating periosteal cells with FGL2 upregulates p-S6 in the mTORC1 signaling pathway, increases glucose uptake, and enhances cell proliferation. However, this effect is abolished under high glucose conditions. Recent studies have demonstrated that FGL2 interacts with the mitochondrial regulator PGC-1α and the energy-sensing protein TMEM120A [38]. These findings suggest that FGL2, as a secreted protein, plays a crucial role in enhancing glucose metabolism during intramembranous bone healing.

Our study employed the drill hole model, which primarily represents intramembranous ossification. However, fractures typically involve a combination of intramembranous and endochondral ossification processes. To better simulate the complexity of bone healing, future experiments should include models such as the tibia fracture model. While we identified the role of the highly expressed secreted protein FGL2 in promoting periosteal cell proliferation, it is possible that other secreted proteins also contribute to this process. Additionally, further experiments are needed to identify the main cell populations responsible for secreting FGL2 and to explore the potential roles of other secreted proteins in periosteal cell proliferation and bone healing.

In summary, our study highlights the complex interplay between diabetes-induced metabolic changes and bone healing processes. Creating a periosteum atlas offers a comprehensive view of cellular changes related to delayed intramembranous bone healing in T2DM. By integrating data from tibial fractures, we can distinguish the intramembranous from endochondral ossification during bone repair. Specifically for T2DM, targeting the metabolic pathways of FAPs and osteogenic cells may present new therapeutic targets. The proliferation of cells in both the inner and outer layers of the periosteum, as well as the factors they secrete, may affect the speed of intramembranous bone healing. Thus, it may be important to not only intervene in the formation of the periosteum but also to consider the impact of the factors they secrete on the healing process. Conclusively, this research provides a nuanced understanding of the interplay between T2DM-induced metabolic derangements and the osseous repair cascade.

### Limitations and future directions

While our study provides valuable insights into the role of the periosteum in T2DM-related bone healing, several limitations should be acknowledged. First, the db/db mouse model, although widely used, does not fully recapitulate the complexity of human T2DM. Future studies should consider using additional models, such as high-fat diet-induced T2DM, to validate our findings. Second, although we identified FGL2 as a key regulator of periosteal cell proliferation, the precise molecular mechanisms remain to be fully elucidated. Future experiments, including genetic knockout and overexpression studies, will be necessary to further dissect the role of FGL2 in bone healing.

## Supplementary Information

Supplementary Material 1.

Supplementary Material 2: Figure S1. Reduced bone mass in 12-week-old db/db mice. A. Body weight (left) and fasting blood glucose (right) of db/db and db/+ mice at different age. One-way ANOVA, Sidak’s multiple comparison test. Data are shown as mean ± SD. *n* = 6 (db/+ and db/db, 8- and 12-week-old); *n* = 3 (db/+ and db/db, 16-week-old). B. Representative stereomicroscopy (left) and 3D μCT images (right) of femurs from db/db and db/+ mice at 12-week-old. Scale bar: 2 mm. C. Femur length of the db/db and db/+ mice at 12- week-old age. Two-tailed Student’s t-test. Data are shown as mean ± SD. *n* = 6 (db/+); *n* = 8 (db/db). D. 3D in vivo μCT images of 1 mm cortical bone segments in the mid-diaphysis of the db/db and db/+ femurs at different ages. Scale bar: 2 mm. E. cortical bone area (Ct.ar), total tissue area (Tt.ar) and cortical thickness (Ct.th) of 1 mm cortical bone segments in the mid-diaphysis of the db/db and db/+ femurs at different ages. Two-way ANOVA, Sidak’s multiple comparison test. Data are shown as mean ± SD. *n* = 3. F. Hematoxylin and eosin staining of the distal femurs from db/db and db/+ mice. Scale bar: 1 mm. *P < 0.05, **P < 0.01, ***P < 0.001. Figure S2. Flow Cytometry and Gene Expression Analysis of Periosteal Cells. A. The flow cytometry gating strategy involved the use of an anti-CD45 antibody to exclude hematopoietic lineage cells. B-I. Vlnplot displaying the expression of key marker genes across different periosteal cell subpopulations identified in the analysis. Subpopulations include proliferating cells, endothelial cells (ECs), Schwann cells, chondrocytes, muscle satellite cells (MuSCs), pericytes, myocytes, IECs and tenocytes. J. UMAP for periosteum from db/+ and db/db. Figure S3. Time-Dependent Changes in the Periosteum Following Drill Hole Surgery. A-F. Panels A to F show the sequential changes in tibias at day 0, 3, 5, 7, 14, and 21 post-drill hole surgery, including: Representative 2D μCT images of horizontal sections of the tibias. Scale bar: 1 mm; Corresponding 3D μCT images of the regions of interest. Scale bar: 500 μm; Hematoxylin and eosin staining; Masson staining; Safranin O staining; Tartrate-resistant acid phosphatase staining. Scale bar: 500 μm and 200 μm G. Quantification of newly forming bone mass at the fracture sites as shown in the 3D μCT images, at 0, 3, 5, 7, 14, and 21 days post-fracture. Statistical analysis was performed using one-way ANOVA with Sidak’s multiple comparison test. Data are presented as mean ± SD, with *n* = 6. H. Representative 3D μCT images of fractured tibias at different time points post-surgery. Scale bar: 1 mm. Figure S4. Impaired callus remodeling and decreased osteogenic activity in db/db mice during fracture healing. A. Representative micro-CT images of fracture callus at day 5 and day 7 post-fracture in db/+ and db/db mice. Scale bars: 500 µm. B. H&E-stained histological sections of fracture callus at day 14 and day 21 post-fracture. Scale bars: 100 µm. C. Representative dynamic bone formation images using double labeling with alizarin red (red) and calcein (green) in callus regions at day 7 post-fracture. D. Quantification of mineral apposition rate (MAR, µm/day) Data are shown as mean ± SD. ***p < 0.001. E. TRAP-stained sections at day 7 post-fracture showing osteoclasts in callus area. F. Quantification of osteoclast surface per bone surface (Oc.S/BS, %). Data are shown as mean ± SD. ***p < 0.001. Figure S5. Cell Type Composition and Marker Gene Expression in Periosteal Cells. A-G. Vlnplot displaying the expression levels of selected marker genes across various periosteal cell subpopulations. Subpopulations include muscle satellite cells (MuSCs), Pericytes, Schwann cells, Proliferating cells, tenocytes, Endothelial cells (ECs), Chondrocytes. Figure S6. Integrated Single-Cell RNA-Seq Analysis of Periosteal Cells Across Multiple Experimental Conditions. A. UMAP visualizations of unsupervised clustering of periosteal cells from various conditions, including db/+, db/+ injured, db/db, db/db injured, Col2-Cre, Col2-Cre fracture Day 5, Col2-Cre fracture Day 10. Clusters are color-coded and labeled, showing distinct subpopulations across different experimental conditions. B. Heatmap displaying differential gene expression across the identified periosteal cell clusters from the UMAP analysis. Key marker genes associated with specific cell types or functions are highlighted on the right. Figure S7. scRNA-Seq Analysis of Distinct Cellular Dynamics in Bone Repair: Comparing Intramembranous and Endochondral Ossification. A-G. Top: UMAP visualizations of unsupervised clustering of periosteal cells from various conditions, including db/+, db/+ injured, db/db, db/db injured, Col2-Cre, Col2-Cre fracture Day 5, Col2-Cre fracture Day 10, Clusters are color-coded and labeled. H. Dot plot showing the expression levels of key marker genes across different cell types, including FAPs, injury induced fibroblasts (IIFCs), chondrocytes (CHs), PCs, and proliferating cell cluster (Mki67^+^). I-K. Feature plots showing the expression of representative marker genes (*Acan*, *Postn*, and *Acta2*) across the UMAP space, highlighting distinct gene expression patterns. Figure S8. Effect of FGL2 on Osteoblast Proliferation Under Normal and High Glucose Conditions. A. Osteoblasts were treated with vehicle or varying concentrations of FGL2 (50, 200 ng/mL) under normal (vehicle) and high glucose (4.5 mg/mL) conditions, and relative cell proliferation was assessed after 48 h to compare the effects under these different conditions. Statistical significance was assessed using one-way ANOVA with Sidak’s multiple comparison test. Data are shown as mean ± SD. **P < 0.01, ***P < 0.001. B. RT-qPCR results showing the mRNA levels of Raptor following knockout using CRISPR-Cas9 lentivirus. Statistical significance was determined using a two-tailed Student’s t-test. Data are shown as mean ± SD. **P < 0.01.

## Data Availability

Single cell transcriptomics data is available in the GEO database (GSE281902).
