# Supplementary material for: Single-cell profiling reveals periosteal signatures of impaired periosteal cells proliferation in a drill-hole model of type 2 diabetes
Source: Cell Commun Signal. 2025 Aug 12;23:371. doi: 10.1186/s12964-025-02349-y (PMC12341304; doi:10.1186/s12964-025-02349-y)
Supplement: Supplementary file 1 — Supplementary Material 1. [file 12964_2025_2349_MOESM1_ESM.zip › revised_Supplemental tables 1.docx]

| Target | Sequence(5’ to 3’) |
| --- | --- |
| Sg-control | CACCGACACGCGCTTCCGCGGCCCGTTCAA |
|  | AAACTTGAACGGGCCGCGGAAGCGCGTGTC |
| Sg-Raptor | CACCGGACGGCTCGGATGTTCCCATAGG |
|  | AAACCCTATGGGAACATCCGAGCCGTCC |

Supplementary Table 1. Oligos for constructing the CRISPR-Cas9 plasmids

| Target | Sequence(5’ to 3’) |
| --- | --- |
| β-actin | ATGGATGACGATATCGCTGC |
|  | CCTGGATGGCTACGTACATG |
| Raptor | CAGTCGCCTCTTATGGGACTC |
|  | GGAGCCTTCGATTTTCTCACA |

Supplementary Table 2. Primers for real-time quantitative PCR
