# Supplementary figures and images for: Single-cell profiling reveals periosteal signatures of impaired periosteal cells proliferation in a drill-hole model of type 2 diabetes
Source: Cell Commun Signal. 2025 Aug 12;23:371. doi: 10.1186/s12964-025-02349-y (PMC12341304; doi:10.1186/s12964-025-02349-y)

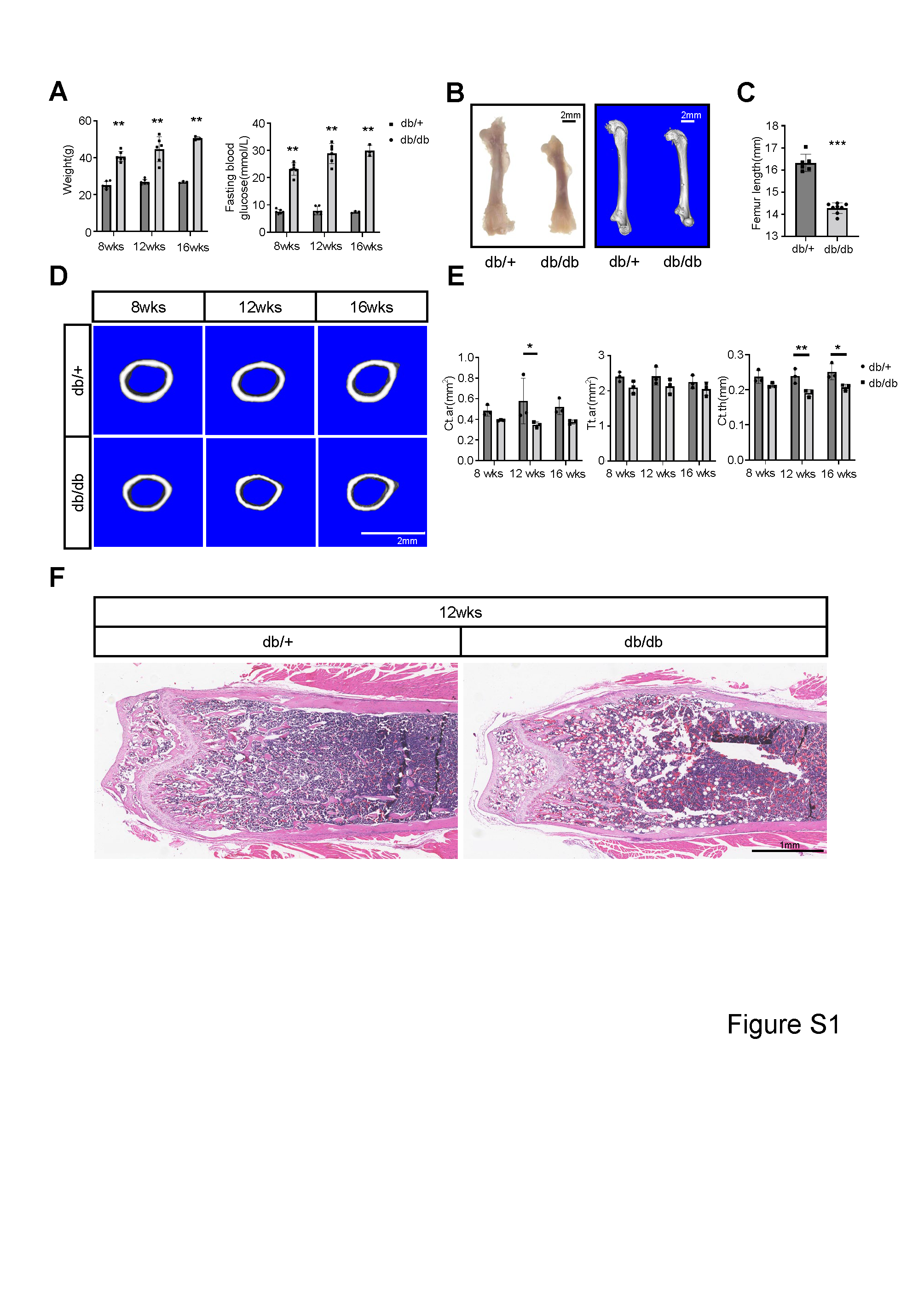

Supplement: Supplementary file 2 — Supplementary Material 2: Figure S1. Reduced bone mass in 12-week-old db/db mice. A. Body weight (left) and fasting blood glucose (right) of db/db and db/+ mice at different age. One-way ANOVA, Sidak’s multiple comparison test. Data are shown as mean ± SD. n = 6 (db/+ and db/db, 8- and 12-week-old); n = 3 (db/+ and db/db, 16-week-old). B. Representative stereomicroscopy (left) and 3D μCT images (right) of femurs from db/db and db/+ mice at 12-week-old. Scale bar: 2 mm. C. Femur length of the db/db and db/+ mice at 12- week-old age. Two-tailed Student’s t-test. Data are shown as mean ± SD. n = 6 (db/+); n = 8 (db/db). D. 3D in vivo μCT images of 1 mm cortical bone segments in the mid-diaphysis of the db/db and db/+ femurs at different ages. Scale bar: 2 mm. E. cortical bone area (Ct.ar), total tissue area (Tt.ar) and cortical thickness (Ct.th) of 1 mm cortical bone segments in the mid-diaphysis of the db/db and db/+ femurs at different ages. Two-way ANOVA, Sidak’s multiple comparison test. Data are shown as mean ± SD. n = 3. F. Hematoxylin and eosin staining of the distal femurs from db/db and db/+ mice. Scale bar: 1 mm. *P < 0.05, **P < 0.01, ***P < 0.001. Figure S2. Flow Cytometry and Gene Expression Analysis of Periosteal Cells. A. The flow cytometry gating strategy involved the use of an anti-CD45 antibody to exclude hematopoietic lineage cells. B-I. Vlnplot displaying the expression of key marker genes across different periosteal cell subpopulations identified in the analysis. Subpopulations include proliferating cells, endothelial cells (ECs), Schwann cells, chondrocytes, muscle satellite cells (MuSCs), pericytes, myocytes, IECs and tenocytes. J. UMAP for periosteum from db/+ and db/db. Figure S3. Time-Dependent Changes in the Periosteum Following Drill Hole Surgery. A-F. Panels A to F show the sequential changes in tibias at day 0, 3, 5, 7, 14, and 21 post-drill hole surgery, including: Representative 2D μCT images of horizontal sections of the tibias. Scale b [file 12964_2025_2349_MOESM2_ESM.zip › Supplemental Figures_1.tif]

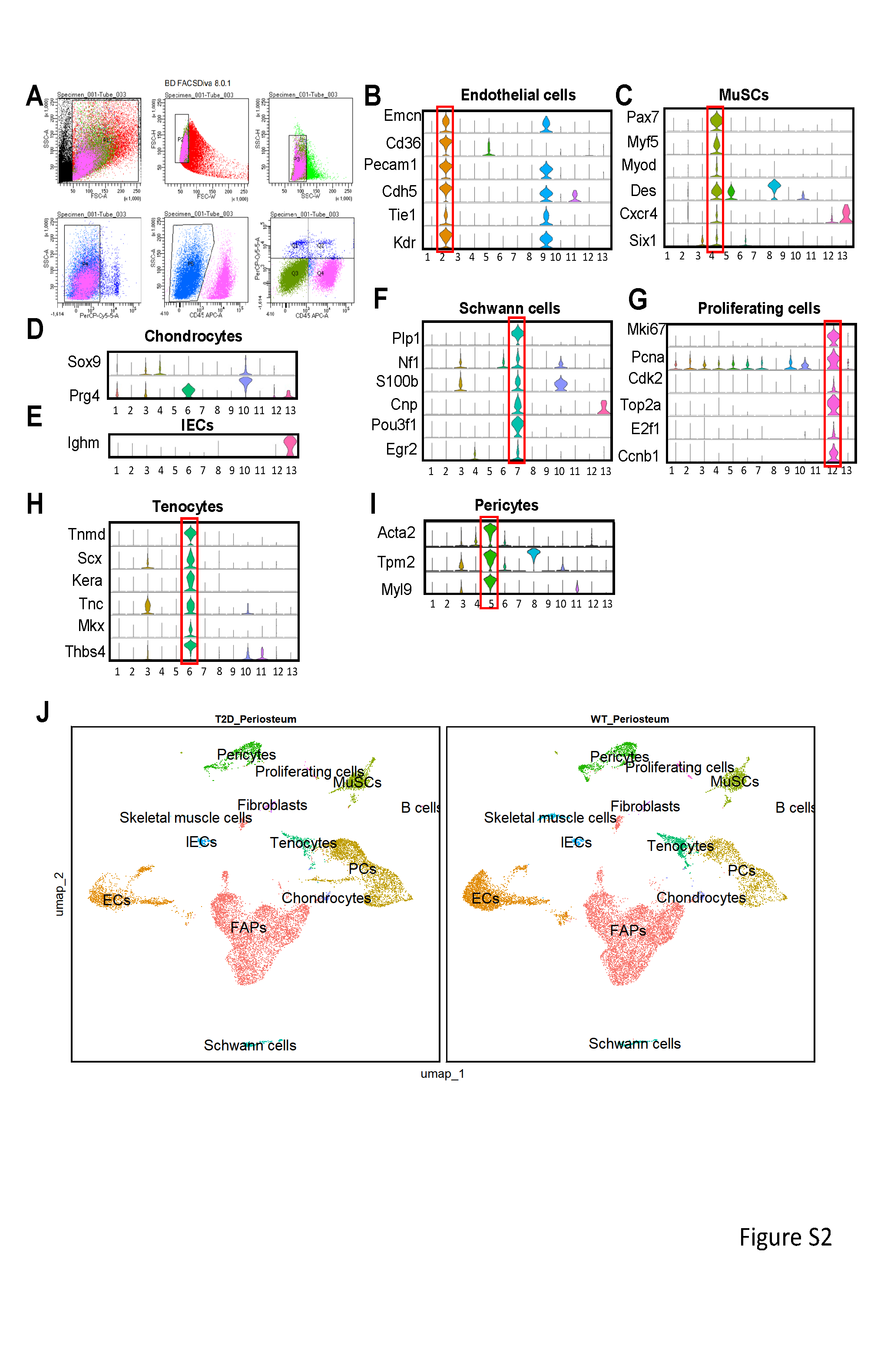

Supplement: Supplementary file 2 — Supplementary Material 2: Figure S1. Reduced bone mass in 12-week-old db/db mice. A. Body weight (left) and fasting blood glucose (right) of db/db and db/+ mice at different age. One-way ANOVA, Sidak’s multiple comparison test. Data are shown as mean ± SD. n = 6 (db/+ and db/db, 8- and 12-week-old); n = 3 (db/+ and db/db, 16-week-old). B. Representative stereomicroscopy (left) and 3D μCT images (right) of femurs from db/db and db/+ mice at 12-week-old. Scale bar: 2 mm. C. Femur length of the db/db and db/+ mice at 12- week-old age. Two-tailed Student’s t-test. Data are shown as mean ± SD. n = 6 (db/+); n = 8 (db/db). D. 3D in vivo μCT images of 1 mm cortical bone segments in the mid-diaphysis of the db/db and db/+ femurs at different ages. Scale bar: 2 mm. E. cortical bone area (Ct.ar), total tissue area (Tt.ar) and cortical thickness (Ct.th) of 1 mm cortical bone segments in the mid-diaphysis of the db/db and db/+ femurs at different ages. Two-way ANOVA, Sidak’s multiple comparison test. Data are shown as mean ± SD. n = 3. F. Hematoxylin and eosin staining of the distal femurs from db/db and db/+ mice. Scale bar: 1 mm. *P < 0.05, **P < 0.01, ***P < 0.001. Figure S2. Flow Cytometry and Gene Expression Analysis of Periosteal Cells. A. The flow cytometry gating strategy involved the use of an anti-CD45 antibody to exclude hematopoietic lineage cells. B-I. Vlnplot displaying the expression of key marker genes across different periosteal cell subpopulations identified in the analysis. Subpopulations include proliferating cells, endothelial cells (ECs), Schwann cells, chondrocytes, muscle satellite cells (MuSCs), pericytes, myocytes, IECs and tenocytes. J. UMAP for periosteum from db/+ and db/db. Figure S3. Time-Dependent Changes in the Periosteum Following Drill Hole Surgery. A-F. Panels A to F show the sequential changes in tibias at day 0, 3, 5, 7, 14, and 21 post-drill hole surgery, including: Representative 2D μCT images of horizontal sections of the tibias. Scale b [file 12964_2025_2349_MOESM2_ESM.zip › Supplemental Figures_2.tif]

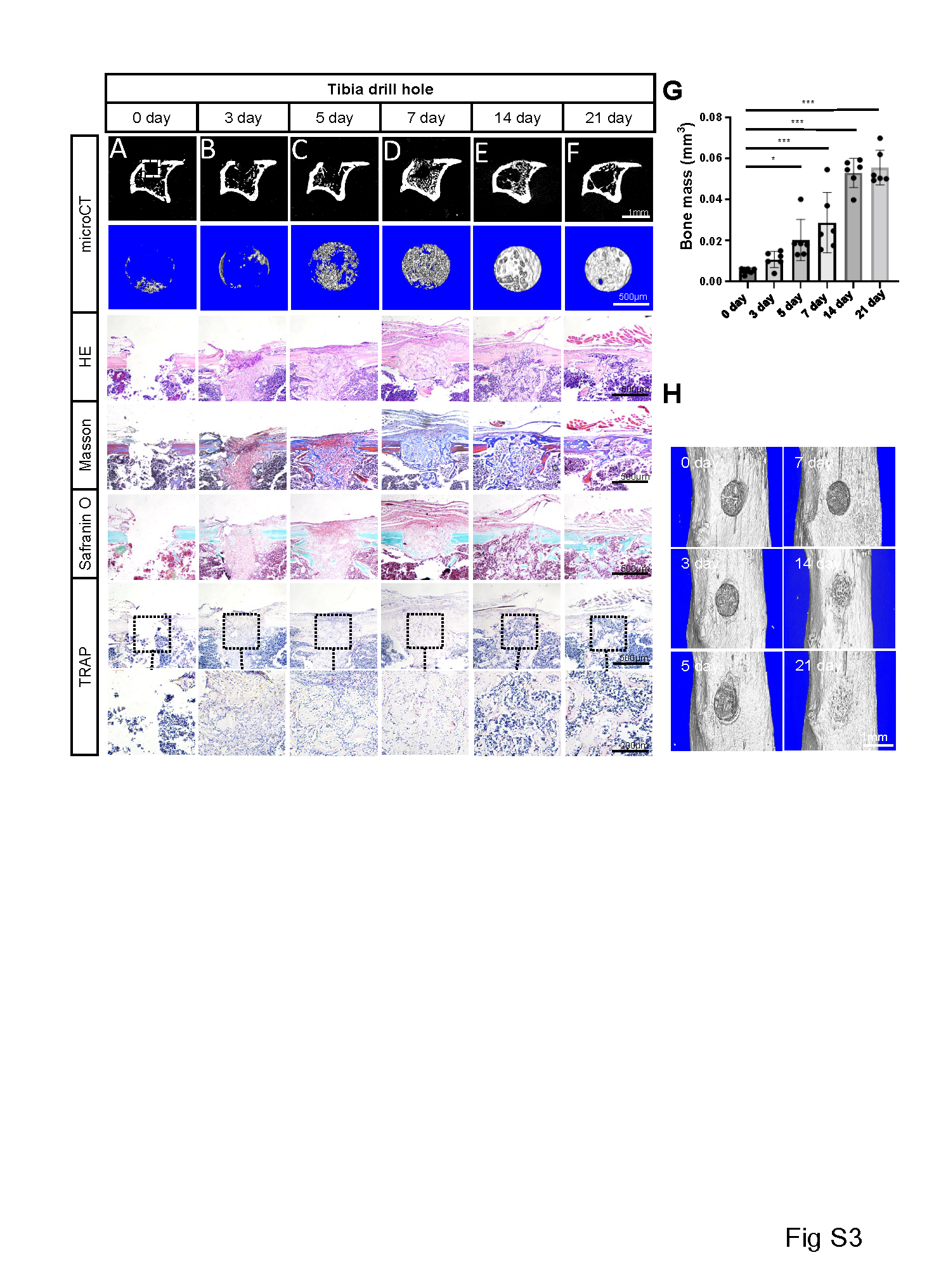

Supplement: Supplementary file 2 — Supplementary Material 2: Figure S1. Reduced bone mass in 12-week-old db/db mice. A. Body weight (left) and fasting blood glucose (right) of db/db and db/+ mice at different age. One-way ANOVA, Sidak’s multiple comparison test. Data are shown as mean ± SD. n = 6 (db/+ and db/db, 8- and 12-week-old); n = 3 (db/+ and db/db, 16-week-old). B. Representative stereomicroscopy (left) and 3D μCT images (right) of femurs from db/db and db/+ mice at 12-week-old. Scale bar: 2 mm. C. Femur length of the db/db and db/+ mice at 12- week-old age. Two-tailed Student’s t-test. Data are shown as mean ± SD. n = 6 (db/+); n = 8 (db/db). D. 3D in vivo μCT images of 1 mm cortical bone segments in the mid-diaphysis of the db/db and db/+ femurs at different ages. Scale bar: 2 mm. E. cortical bone area (Ct.ar), total tissue area (Tt.ar) and cortical thickness (Ct.th) of 1 mm cortical bone segments in the mid-diaphysis of the db/db and db/+ femurs at different ages. Two-way ANOVA, Sidak’s multiple comparison test. Data are shown as mean ± SD. n = 3. F. Hematoxylin and eosin staining of the distal femurs from db/db and db/+ mice. Scale bar: 1 mm. *P < 0.05, **P < 0.01, ***P < 0.001. Figure S2. Flow Cytometry and Gene Expression Analysis of Periosteal Cells. A. The flow cytometry gating strategy involved the use of an anti-CD45 antibody to exclude hematopoietic lineage cells. B-I. Vlnplot displaying the expression of key marker genes across different periosteal cell subpopulations identified in the analysis. Subpopulations include proliferating cells, endothelial cells (ECs), Schwann cells, chondrocytes, muscle satellite cells (MuSCs), pericytes, myocytes, IECs and tenocytes. J. UMAP for periosteum from db/+ and db/db. Figure S3. Time-Dependent Changes in the Periosteum Following Drill Hole Surgery. A-F. Panels A to F show the sequential changes in tibias at day 0, 3, 5, 7, 14, and 21 post-drill hole surgery, including: Representative 2D μCT images of horizontal sections of the tibias. Scale b [file 12964_2025_2349_MOESM2_ESM.zip › Supplemental Figures_revised_3.tif]

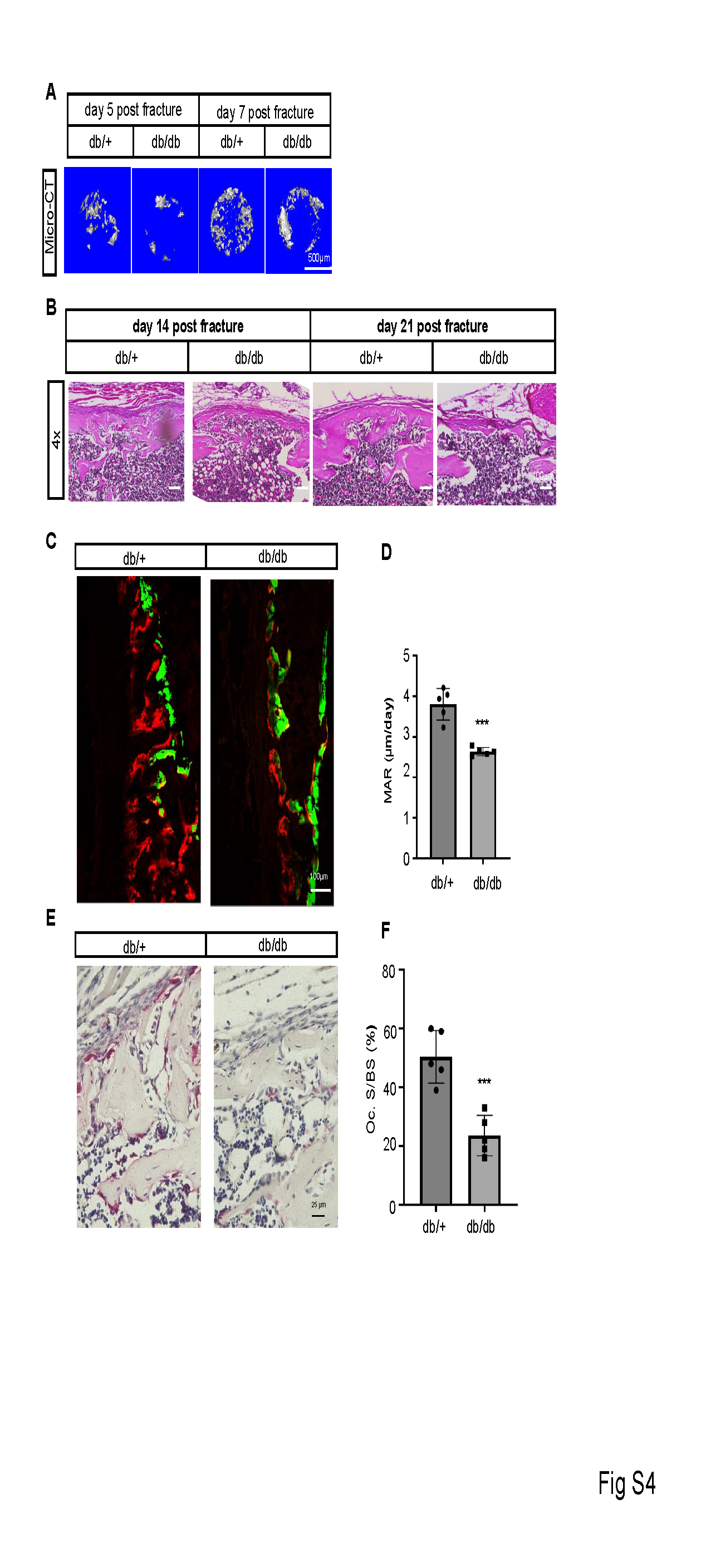

Supplement: Supplementary file 2 — Supplementary Material 2: Figure S1. Reduced bone mass in 12-week-old db/db mice. A. Body weight (left) and fasting blood glucose (right) of db/db and db/+ mice at different age. One-way ANOVA, Sidak’s multiple comparison test. Data are shown as mean ± SD. n = 6 (db/+ and db/db, 8- and 12-week-old); n = 3 (db/+ and db/db, 16-week-old). B. Representative stereomicroscopy (left) and 3D μCT images (right) of femurs from db/db and db/+ mice at 12-week-old. Scale bar: 2 mm. C. Femur length of the db/db and db/+ mice at 12- week-old age. Two-tailed Student’s t-test. Data are shown as mean ± SD. n = 6 (db/+); n = 8 (db/db). D. 3D in vivo μCT images of 1 mm cortical bone segments in the mid-diaphysis of the db/db and db/+ femurs at different ages. Scale bar: 2 mm. E. cortical bone area (Ct.ar), total tissue area (Tt.ar) and cortical thickness (Ct.th) of 1 mm cortical bone segments in the mid-diaphysis of the db/db and db/+ femurs at different ages. Two-way ANOVA, Sidak’s multiple comparison test. Data are shown as mean ± SD. n = 3. F. Hematoxylin and eosin staining of the distal femurs from db/db and db/+ mice. Scale bar: 1 mm. *P < 0.05, **P < 0.01, ***P < 0.001. Figure S2. Flow Cytometry and Gene Expression Analysis of Periosteal Cells. A. The flow cytometry gating strategy involved the use of an anti-CD45 antibody to exclude hematopoietic lineage cells. B-I. Vlnplot displaying the expression of key marker genes across different periosteal cell subpopulations identified in the analysis. Subpopulations include proliferating cells, endothelial cells (ECs), Schwann cells, chondrocytes, muscle satellite cells (MuSCs), pericytes, myocytes, IECs and tenocytes. J. UMAP for periosteum from db/+ and db/db. Figure S3. Time-Dependent Changes in the Periosteum Following Drill Hole Surgery. A-F. Panels A to F show the sequential changes in tibias at day 0, 3, 5, 7, 14, and 21 post-drill hole surgery, including: Representative 2D μCT images of horizontal sections of the tibias. Scale b [file 12964_2025_2349_MOESM2_ESM.zip › Supplemental Figures_revised_4.tif]

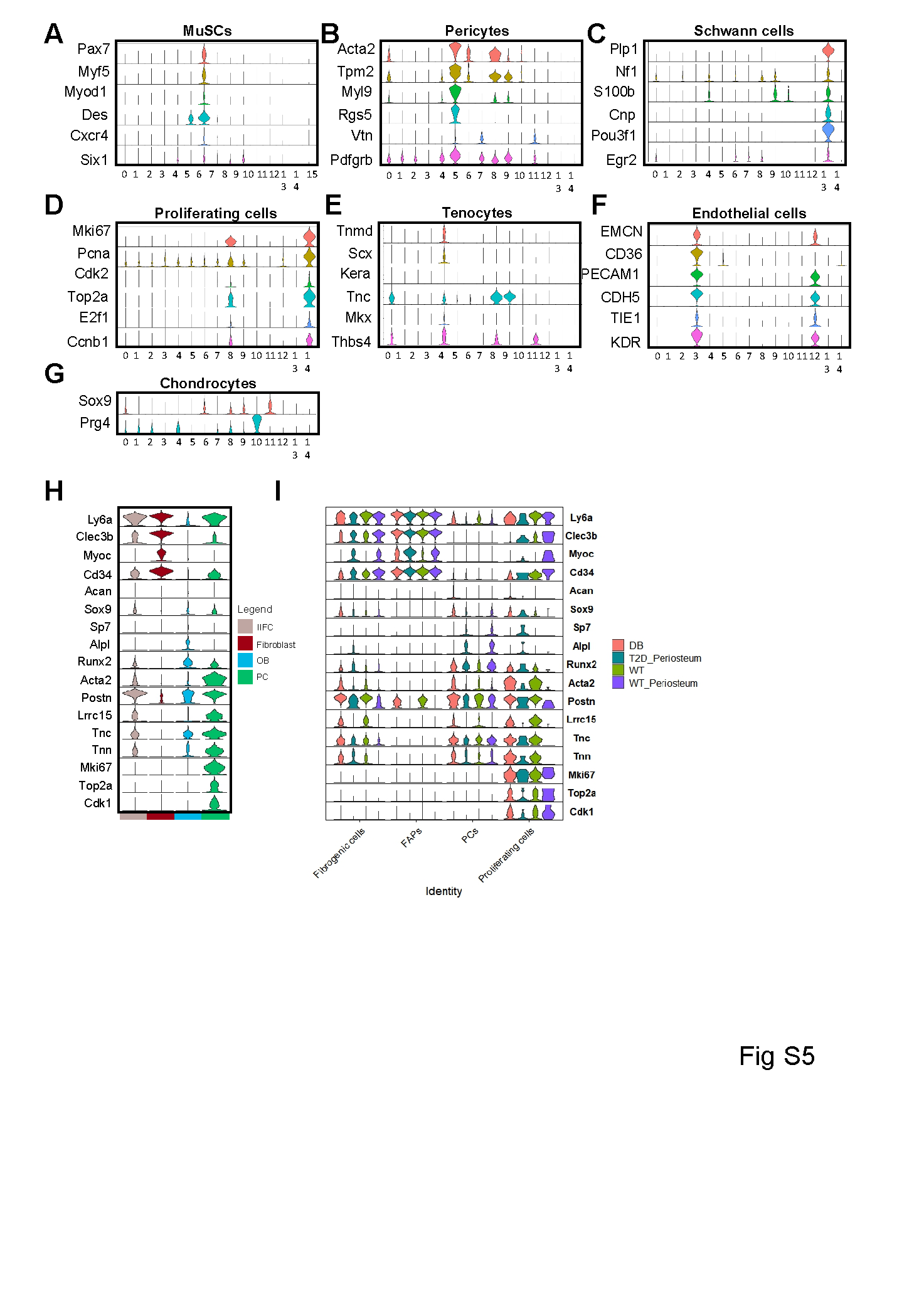

Supplement: Supplementary file 2 — Supplementary Material 2: Figure S1. Reduced bone mass in 12-week-old db/db mice. A. Body weight (left) and fasting blood glucose (right) of db/db and db/+ mice at different age. One-way ANOVA, Sidak’s multiple comparison test. Data are shown as mean ± SD. n = 6 (db/+ and db/db, 8- and 12-week-old); n = 3 (db/+ and db/db, 16-week-old). B. Representative stereomicroscopy (left) and 3D μCT images (right) of femurs from db/db and db/+ mice at 12-week-old. Scale bar: 2 mm. C. Femur length of the db/db and db/+ mice at 12- week-old age. Two-tailed Student’s t-test. Data are shown as mean ± SD. n = 6 (db/+); n = 8 (db/db). D. 3D in vivo μCT images of 1 mm cortical bone segments in the mid-diaphysis of the db/db and db/+ femurs at different ages. Scale bar: 2 mm. E. cortical bone area (Ct.ar), total tissue area (Tt.ar) and cortical thickness (Ct.th) of 1 mm cortical bone segments in the mid-diaphysis of the db/db and db/+ femurs at different ages. Two-way ANOVA, Sidak’s multiple comparison test. Data are shown as mean ± SD. n = 3. F. Hematoxylin and eosin staining of the distal femurs from db/db and db/+ mice. Scale bar: 1 mm. *P < 0.05, **P < 0.01, ***P < 0.001. Figure S2. Flow Cytometry and Gene Expression Analysis of Periosteal Cells. A. The flow cytometry gating strategy involved the use of an anti-CD45 antibody to exclude hematopoietic lineage cells. B-I. Vlnplot displaying the expression of key marker genes across different periosteal cell subpopulations identified in the analysis. Subpopulations include proliferating cells, endothelial cells (ECs), Schwann cells, chondrocytes, muscle satellite cells (MuSCs), pericytes, myocytes, IECs and tenocytes. J. UMAP for periosteum from db/+ and db/db. Figure S3. Time-Dependent Changes in the Periosteum Following Drill Hole Surgery. A-F. Panels A to F show the sequential changes in tibias at day 0, 3, 5, 7, 14, and 21 post-drill hole surgery, including: Representative 2D μCT images of horizontal sections of the tibias. Scale b [file 12964_2025_2349_MOESM2_ESM.zip › Supplemental Figures_revised_5.tif]

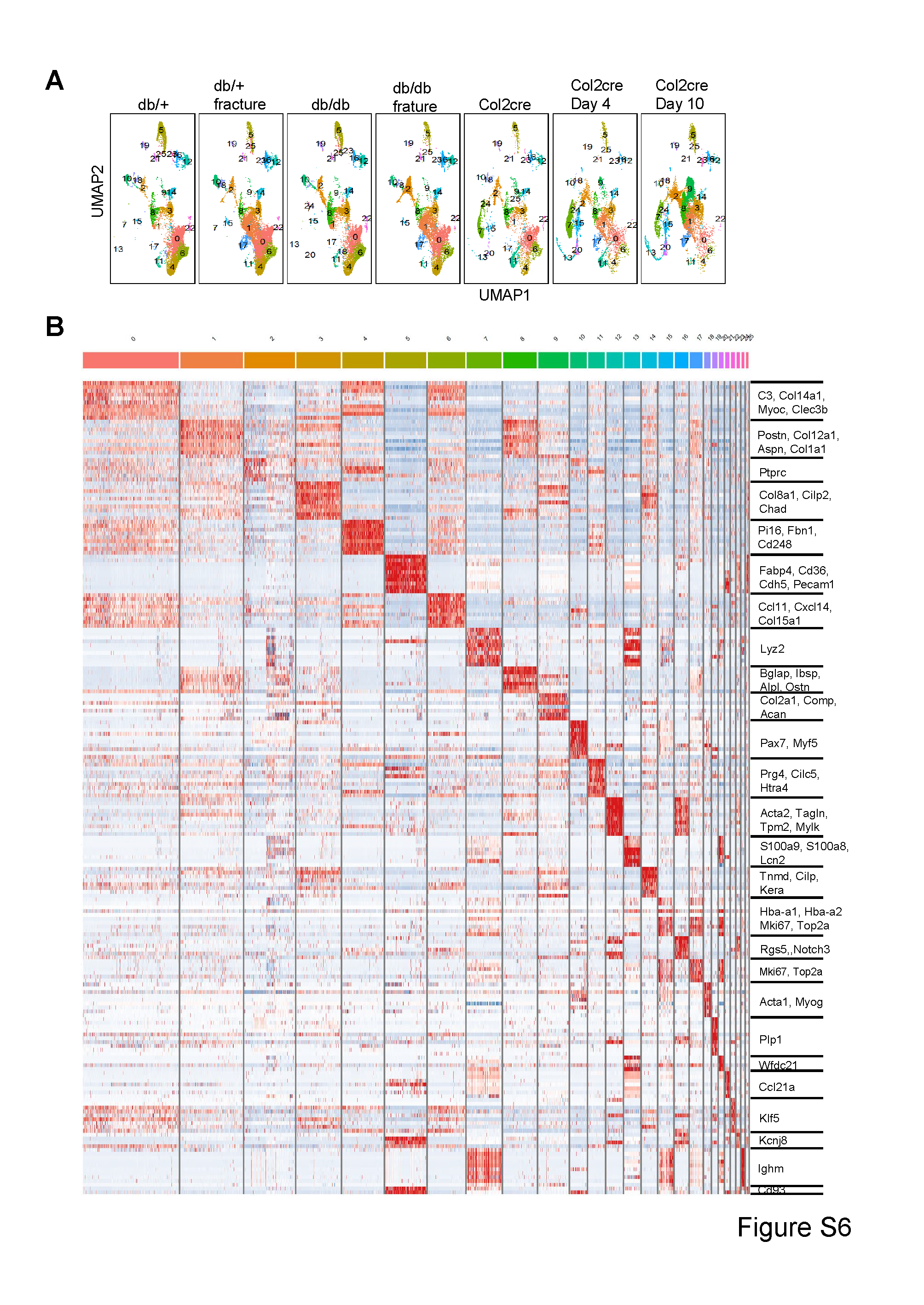

Supplement: Supplementary file 2 — Supplementary Material 2: Figure S1. Reduced bone mass in 12-week-old db/db mice. A. Body weight (left) and fasting blood glucose (right) of db/db and db/+ mice at different age. One-way ANOVA, Sidak’s multiple comparison test. Data are shown as mean ± SD. n = 6 (db/+ and db/db, 8- and 12-week-old); n = 3 (db/+ and db/db, 16-week-old). B. Representative stereomicroscopy (left) and 3D μCT images (right) of femurs from db/db and db/+ mice at 12-week-old. Scale bar: 2 mm. C. Femur length of the db/db and db/+ mice at 12- week-old age. Two-tailed Student’s t-test. Data are shown as mean ± SD. n = 6 (db/+); n = 8 (db/db). D. 3D in vivo μCT images of 1 mm cortical bone segments in the mid-diaphysis of the db/db and db/+ femurs at different ages. Scale bar: 2 mm. E. cortical bone area (Ct.ar), total tissue area (Tt.ar) and cortical thickness (Ct.th) of 1 mm cortical bone segments in the mid-diaphysis of the db/db and db/+ femurs at different ages. Two-way ANOVA, Sidak’s multiple comparison test. Data are shown as mean ± SD. n = 3. F. Hematoxylin and eosin staining of the distal femurs from db/db and db/+ mice. Scale bar: 1 mm. *P < 0.05, **P < 0.01, ***P < 0.001. Figure S2. Flow Cytometry and Gene Expression Analysis of Periosteal Cells. A. The flow cytometry gating strategy involved the use of an anti-CD45 antibody to exclude hematopoietic lineage cells. B-I. Vlnplot displaying the expression of key marker genes across different periosteal cell subpopulations identified in the analysis. Subpopulations include proliferating cells, endothelial cells (ECs), Schwann cells, chondrocytes, muscle satellite cells (MuSCs), pericytes, myocytes, IECs and tenocytes. J. UMAP for periosteum from db/+ and db/db. Figure S3. Time-Dependent Changes in the Periosteum Following Drill Hole Surgery. A-F. Panels A to F show the sequential changes in tibias at day 0, 3, 5, 7, 14, and 21 post-drill hole surgery, including: Representative 2D μCT images of horizontal sections of the tibias. Scale b [file 12964_2025_2349_MOESM2_ESM.zip › Supplemental Figures_revised_6.tif]

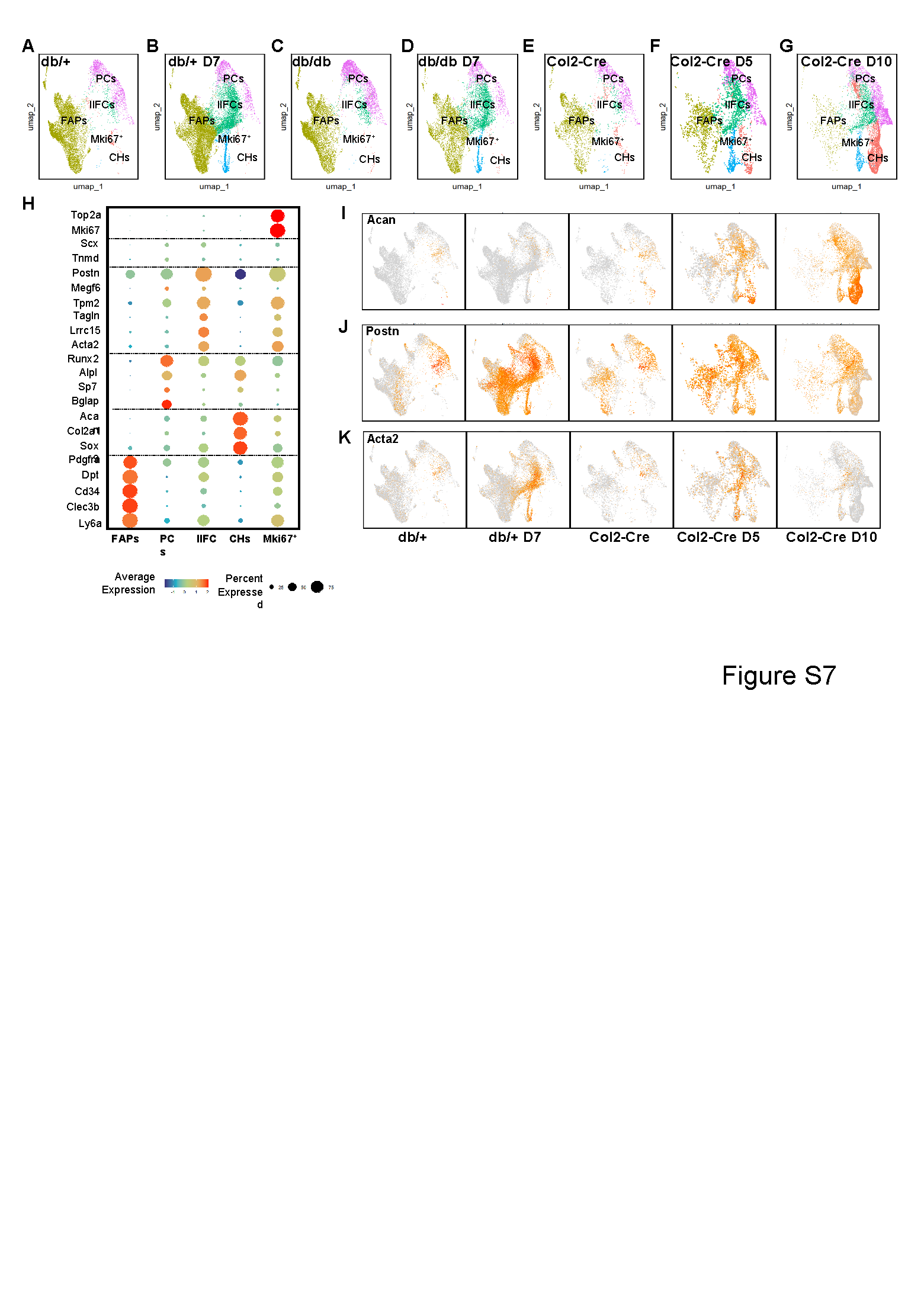

Supplement: Supplementary file 2 — Supplementary Material 2: Figure S1. Reduced bone mass in 12-week-old db/db mice. A. Body weight (left) and fasting blood glucose (right) of db/db and db/+ mice at different age. One-way ANOVA, Sidak’s multiple comparison test. Data are shown as mean ± SD. n = 6 (db/+ and db/db, 8- and 12-week-old); n = 3 (db/+ and db/db, 16-week-old). B. Representative stereomicroscopy (left) and 3D μCT images (right) of femurs from db/db and db/+ mice at 12-week-old. Scale bar: 2 mm. C. Femur length of the db/db and db/+ mice at 12- week-old age. Two-tailed Student’s t-test. Data are shown as mean ± SD. n = 6 (db/+); n = 8 (db/db). D. 3D in vivo μCT images of 1 mm cortical bone segments in the mid-diaphysis of the db/db and db/+ femurs at different ages. Scale bar: 2 mm. E. cortical bone area (Ct.ar), total tissue area (Tt.ar) and cortical thickness (Ct.th) of 1 mm cortical bone segments in the mid-diaphysis of the db/db and db/+ femurs at different ages. Two-way ANOVA, Sidak’s multiple comparison test. Data are shown as mean ± SD. n = 3. F. Hematoxylin and eosin staining of the distal femurs from db/db and db/+ mice. Scale bar: 1 mm. *P < 0.05, **P < 0.01, ***P < 0.001. Figure S2. Flow Cytometry and Gene Expression Analysis of Periosteal Cells. A. The flow cytometry gating strategy involved the use of an anti-CD45 antibody to exclude hematopoietic lineage cells. B-I. Vlnplot displaying the expression of key marker genes across different periosteal cell subpopulations identified in the analysis. Subpopulations include proliferating cells, endothelial cells (ECs), Schwann cells, chondrocytes, muscle satellite cells (MuSCs), pericytes, myocytes, IECs and tenocytes. J. UMAP for periosteum from db/+ and db/db. Figure S3. Time-Dependent Changes in the Periosteum Following Drill Hole Surgery. A-F. Panels A to F show the sequential changes in tibias at day 0, 3, 5, 7, 14, and 21 post-drill hole surgery, including: Representative 2D μCT images of horizontal sections of the tibias. Scale b [file 12964_2025_2349_MOESM2_ESM.zip › Supplemental Figures_revised_7.tif]
